# VirStrain: a strain identification tool for RNA viruses

**DOI:** 10.1186/s13059-022-02609-x

**Published:** 2022-01-31

**Authors:** Herui Liao, Dehan Cai, Yanni Sun

**Affiliations:** Department of Electrical Engineering, City University of Hong Kong, Kowloon, China

**Keywords:** RNA virus, Strain-level analysis, *k*-mer

## Abstract

Viruses change constantly during replication, leading to high intra-species diversity. Although many changes are neutral or deleterious, some can confer on the virus different biological properties such as better adaptability. In addition, viral genotypes often have associated metadata, such as host residence, which can help with inferring viral transmission during pandemics. Thus, subspecies analysis can provide important insights into virus characterization. Here, we present VirStrain, a tool taking short reads as input with viral strain composition as output. We rigorously test VirStrain on multiple simulated and real virus sequencing datasets. VirStrain outperforms the state-of-the-art tools in both sensitivity and accuracy.

**Supplementary Information:**

The online version contains supplementary material available at (10.1186/s13059-022-02609-x).

## Introduction

RNA viruses usually lack strict proofreading mechanisms during replication, leading to new copies containing genetic variations from the parent strains. Many of these variations can be neutral or deleterious to the virus survival. However, some mutations are beneficial to the fitness of the virus [1, 2]. Sequenced viral genomes often have associated metadata, such as the infection time, the host’s residence, gender, ethnicity, and drug usage, which are important to infer the virus evolution and transmission during pandemics [3–5]. Thus, subspecies composition analysis can provide important insights into virus function characterization, viral disease control, and vaccine design.

As pointed out in [6], it is difficult to give a universal definition of microbial strain. Depending on the context, strain can refer to a virus variant with unique and stable phenotypic characteristics under natural conditions [7], or a specific viral genome [3, 6, 8–10]. In this context, strain refers to a specific viral genome.

Recent advances in sequencing technologies enable researchers to conduct subspecies-level composition analysis with unprecedented resolution. Both whole genome sequencing and metagenomic sequencing have been conducted extensively for subspecies virus analysis. Strain-level composition analysis has been intensively studied for bacteria. There are a plethora of tools for identifying bacterial strains from metagenomic data [6]. The available tools are divided into four groups based on their utilities. One group contains tools for known strain identification [9, 11], which takes the reference strain database and short reads as input and outputs the composition analysis of the known strains in the sequenced sample. As known strains often have annotated information such as the subtypes, clades, and other biological properties, this type of tools can provide important insights into the downstream analysis of the microbial communities.

For viruses, besides the annotated phenotypes associated with clades, subtypes, and strains, the strain-associated metadata such as symptoms, geographical location, and travel history also makes known strain identification useful for inferring the transmission path when the contact history is ambiguous or missing. Studies that used the strain genomes for studying the spread of COVID-19 showed that the clusters of the reference genomes are highly structured and are consistent with their geographical distributions [5].

The difficulty of strain identification stems from high similarity between strain genomes. The viral strains with different biological properties may still share very high sequence similarities. For example, Alpha, Beta, Gamma, and Delta strains of SARS-CoV-2 have different transmissibility, disease severity, and risk of reinfection. But they are >99.5*%* identical and have about 50 to 70 mutations in their genomes. If using read mapping for known strain identification, short reads tend to ambiguously map or align to multiple reference genomes. Dissolving the ambiguity in the alignment is computationally expensive [9]. Faster methods often cannot distinguish highly similar strains [12–14] or they have to sacrifice the resolution by only keeping reference genomes with similarity below a given cutoff. In addition, RNA viral strain identification tools should be able to detect more than one strain if there are multi-strain infections, which is not rare for RNA viral diseases. The available tools often have various limitations in strain-level analysis. We summarize related work in the following section.

### Related work

When near-complete virus genomes can be assembled from the sequencing data, alignment-based tools such as BLAST can be applied to find reference genomes that best match the input sequence. However, short contigs or reads tend to align to multiple reference genomes when employing alignment-based tools, such that determining a best match proves difficult. Available tools and websites that can monitor the mutations in strain genomes such as NextFlu and NextStrain [3, 15] also take genomes as input and assign the genome into major genetic groups. When there are difficulties in constructing high-quality virus genomes due to complexity of the data (e.g., metagenomic data), the low abundance of the virus, or the presence of a minor strain besides a major one, there is a need for a tool that can still identify the strains using reads as input.

When the goal is bacterial strains, there are some tools for strain-level analysis using short reads as input. The available reference-based strain-level analysis tools were divided into four groups by Yan et al. [6]. The first group focused on identifying known genotypes from reference genomes [9, 11], which are related to our work. The representative tools in this group, PathoScope [11] and Sigma [9], rely on ambiguity-resolved read mapping strategies between short reads and reference genomes with high sequence similarity. Both tools allow users to create their own reference database and thus can be applied to viruses. However, they are too slow for identifying strains with tens of thousands reference genomes and large-scale sequencing data.

Other bacteria-centered tools cannot be conveniently re-purposed for virus strain analysis because they use bacteria-specific features such as bacterial marker genes or structural variants. For example, our experiments showed that existing marker gene sets can recognize HIV if the reads are sequenced from the dominant strain HXB2, but not other strains of HIV [16]. One possible reason is that the marker gene derivation process of the existing programs [17] did not use all the available viral strains.

Using the available haplotypes or strains to infer transmission has been applied to COVID-19. For example, Gudbjartsson et al. [5] are able to assign a sample to its closest haplotype based on a manually derived haplotype table. However, it is not clear the manually created table can scale to larger datasets or other viruses.

A very relevant tool, QuantTB [18], is targeted at identifying individual *M. tuberculosis* strains with high similarity. However, their tool is “hard coded” for *M. tuberculosis* and thus we cannot conveniently extend it to viruses. In addition, they also applied different thresholds on the number of distinct SNPs between strains, which are actually still stringent for newly identified RNA viruses such as SARS-CoV-2.

For RNA viruses, viral haplotype reconstruction is often applied to reconstruct co-infecting viral haplotypes from viral sequencing data [19–23]. Haplotype reconstruction can be divided into two types: reference-based and de novo. Technically speaking, we can apply haplotype reconstruction tools for known strain identification because the reconstructed haplotypes can be reference strains or novel ones. However, haplotype reconstruction from short reads is more challenging than de novo assembly and usually the performance deteriorates with the increased number of haplotypes and their similarities. Thus, although there are a number of haplotype reconstruction tools, they all have their limitations in reconstructing low-abundance strains, producing full-length haplotypes, or distinguishing highly similar haplotypes. A couple of haplotype reconstruction tools such as CliqueSNV [24] can handle highly similar haplotypes but require tremendous amount of computational resources. We will benchmark against several popular haplotype reconstruction tools in our experiments.

## Results

In order to test our tool on viruses with a large number of reference strain genomes and different mutations rates, we mainly assessed our tool on three types of RNA viruses. The first is SARS-CoV-2, many of which have very high sequence similarity and may differ only at a few sites. The second is the “HA” region of Influenza A H1N1, which has lower average similarity than SARS-CoV-2 but higher similarity within the same clades and sub-clades. The third is HIV, which has a much lower similarity than SARS-CoV-2 and H1N1. For HIV, we used the “Gag” region, which is one of the marker genes for HIV subtype classification [25, 26]. Although our focus is RNA viruses, VirStrain can also be applied to DNA viruses, which can be much larger than RNA viruses. We will assess VirStrain on mixed strain identification of 2 well-studied DNA viruses, hepatitis B virus (HBV) and human cytomegalovirus (HCMV). The available HBV strains have lower sequence similarity than RNA viruses, forming a good test case for the MSA construction stage of VirStrain. HCMV has genome size of around 236kbps, allowing us to test the scalability of VirStrain.

### Data and clustering results

To construct the reference database for VirStrain, we collected all available complete genomes of SARS-CoV-2 from NCBI, H1N1 (HA) from Influenza Research Database (IRD, http://www.fludb.org), and HIV (Gag) from HIV database (http://www.hiv.lanl.gov) as of July 14, 2020. VirStrain allows the users to construct their own databases by inputting a file containing all reference genomes.

**Fig. 1:**
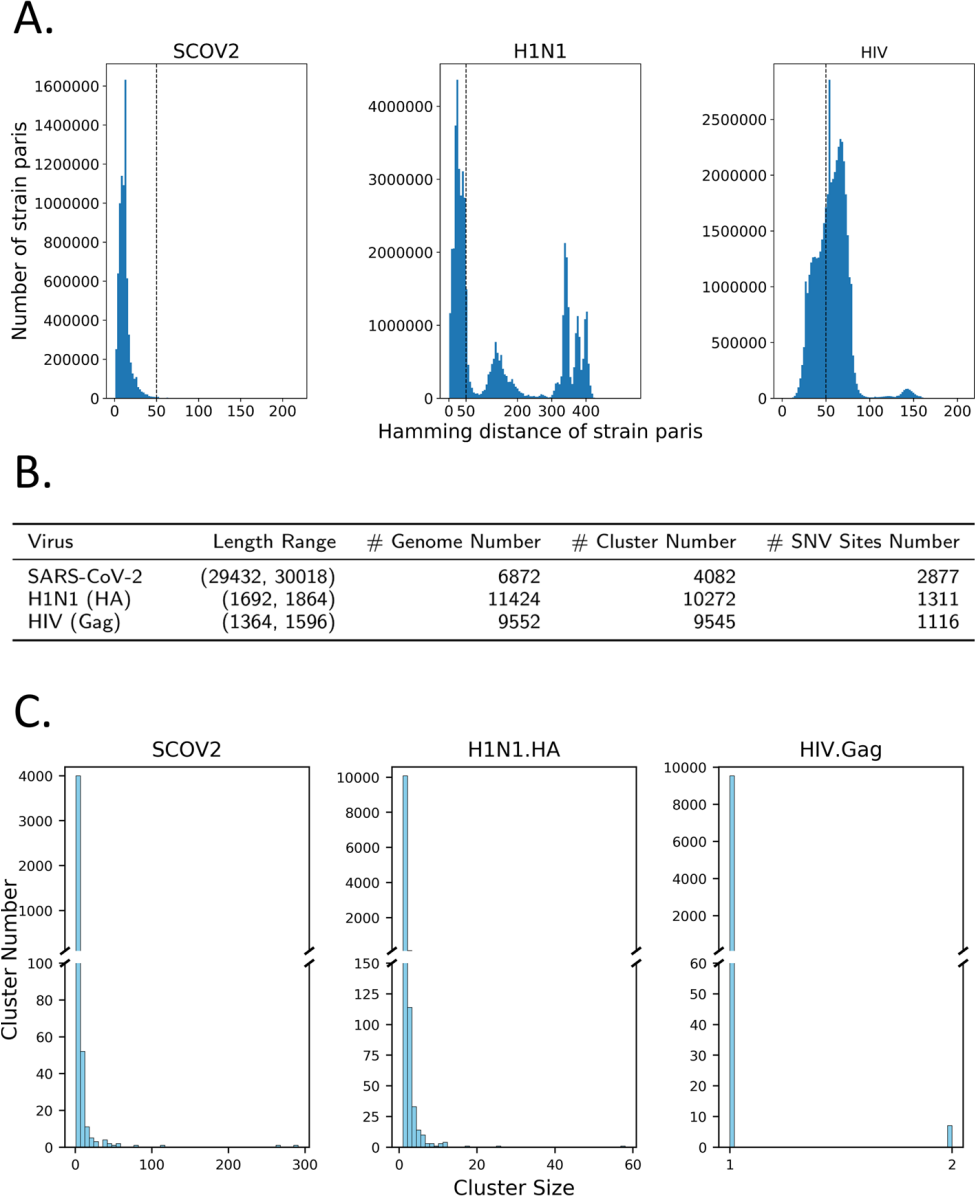
**A** The pairwise Hamming distance distribution of SARS-CoV-2, H1N1 (HA), and HIV (Gag). The Hamming distance here is measured only on the chosen SNV sites by the greedy covering algorithm. They can represent the overall similarity of the three reference sets. The dashed line is at distance value 50 across the three panels for easy comparison. **B** The statistics of the reference database of 3 viruses. “# Genome Number”: the number of downloaded genomes. “# Cluster Number”: the number of final clusters generated by VirStrain. “# SNV Sites Number”: the number of SNV sites chosen by VirStrain for each virus. **C** The cluster size distribution of SARS-CoV-2, H1N1 (HA), and HIV (Gag). “Cluster size” represents the number of strains contained in each cluster

Different metrics are available to quantify the similarity of viral strains [27, 28]. Because our method aligns all the reference strains, it is convenient to directly use pairwise Hamming distance at the SNV sites chosen by VirStrain. Figure 1A compares the pairwise Hamming distance derived from the aligned reference sequences. As expected, SARS-CoV-2 has the highest similarity. H1N1 has multiple peaks showing heterogeneous pairwise strain similarities. HIV is more diverged.

Figure 1B summarizes the statistics of the reference genomes/genes of these three viruses, their final clustering results, and also the number of their SNV sites chosen by VirStrain. It shows that the number of HIV (Gag) sequences is nearly equal to the number of clusters while the other 2 viruses differ a lot, which is caused by relatively low sequence similarity between HIV (Gag).

The cluster size distribution of three viruses are displayed in Fig. 1C. Most clusters are very small, with many containing a single genome. But there are also genomes that cannot be distinguished by the chosen SNV sites. Especially, there are strains with different lengths, leading to alignments with many gaps at the beginning and ending parts. Those columns are not utilized by the greedy covering algorithm. Thus, these strains are usually in the same cluster.

### Overview of the experiments

The input to VirStrain are short reads from either relatively pure or highly mixed samples (such as viral metagenomic data). VirStrain is able to directly return strains from both.

**Fig. 2:**
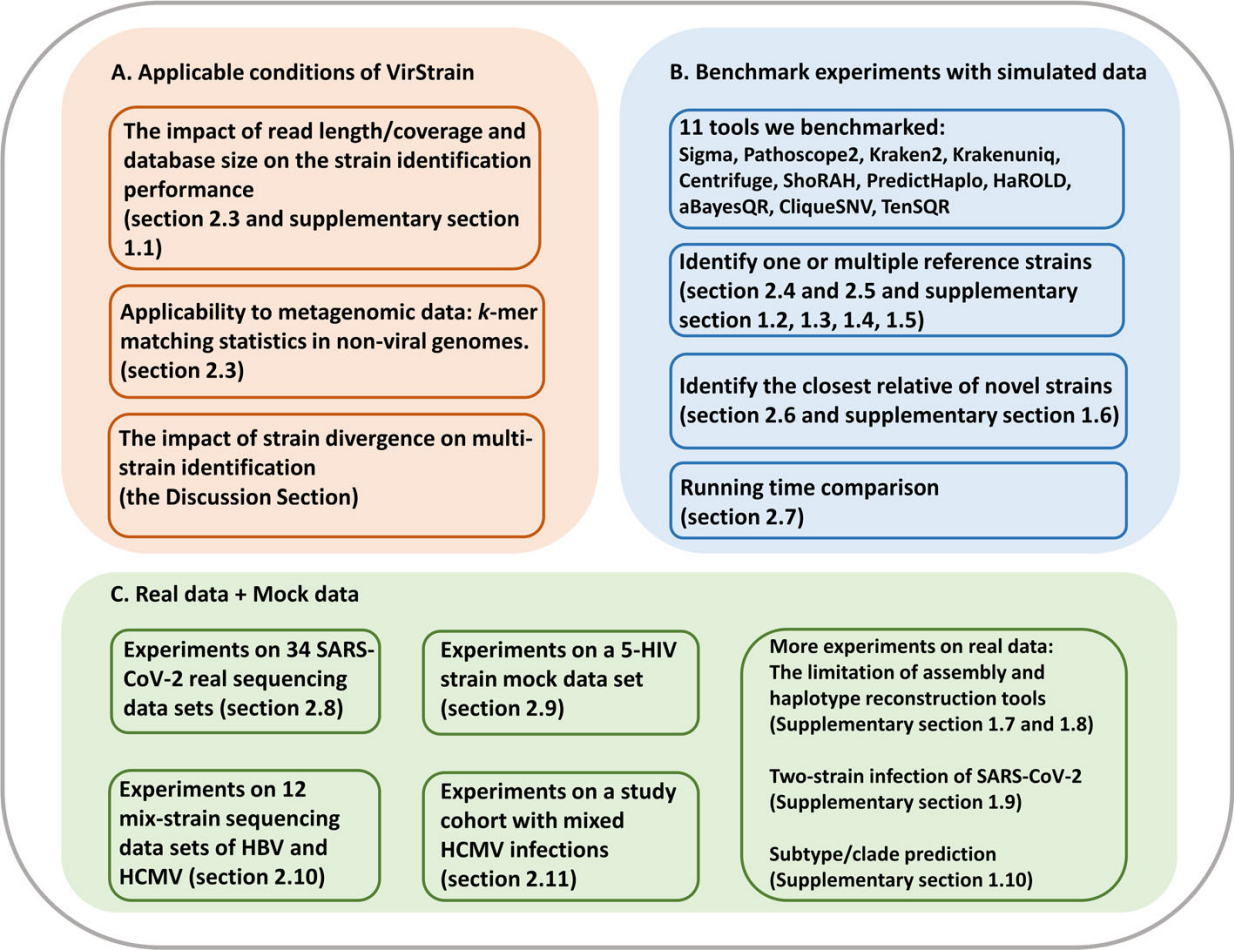
The overview of all experiments

We assessed VirStrain from multiple aspects. The organization of all experiments is summarized in Fig. 2. First, we focused on evaluating the possible limitations of VirStrain based on the method design (Fig. 2A). In particular, we will evaluate how read length, sequencing coverage, and the reference database size affect the performance of VirStrain. Then, we will investigate the applicability of VirStrain to heterogeneous data (e.g., metagenomic data) by conducting *k*-mer match against reads from the host, bacteria, etc. In addition, we will provide some guidance about the acceptable strain divergence in multi-strain detection cases. Second, we benchmarked VirStrain against other popular strain-level analysis tools and haplotype reconstruction tools with simulated data (Fig. 2B). Third, we validated VirStrain in multiple usage scenarios with both real data and mock data (Fig. 2C).

In all these experiments, we use accuracy as the main performance metric for different tools. It is worth noting that all tested tools usually output multiple strains with associated ranking. If we know the number of strains (e.g., x) in a sample, we will keep only the top x outputs of a tool. Then, the *accuracy* is defined as the percentage of correctly identified strains in the output. It is noted that if multiple tied best matches are presented, with the correct strain among them, this will be counted as correct. We will quantify the number of “tie cases” in our experiments.

### Baseline performance of VirStrain

The strain identification performance can be affected by sequencing coverage, read length, similarity of strains, and the size of the reference database. In order to provide users with guidance on utility of VirStrain, we conducted experiments to evaluate the robustness of VirStrain when the input data have different properties. First, we evaluated how the read length, sequencing coverage, and database size affect the performance of VirStrain. Second, we evaluated whether the *k*-mer derived by VirStrain could falsely match other microbes, which is important for applying VirStrain to heterogeneous data such as viral metagenomic data. Third, we focused on evaluating the minimum divergence between the strains for VirStrain to identify them in the multi-strain infection case.

Because SARS-CoV-2 is of high interest and has large size and high strain-level similarity, we conducted all experiments in this section on SARS-CoV-2. As some strains contain non-ACGT characters, we did the experiments by only simulating reads from 2280 SARS-CoV-2 strains that do not contain non-ACGT characters.

#### Impact of read length/sequencing coverage on VirStrain

In order to evaluate the impact of read length and sequencing depth on VirStrain, we simulated reads from each single strain with 5 different sequencing depths and 4 different read lengths. Thus, there are altogether 20 combinations as shown in Table 1. For each combination, we conducted 2280 experiments using ART simulated reads [29] from each of the 2280 strains as input. For all these experiments, we found that the known reference strain always has the best *Vscore* (see the “Step 2: iterative strain search algorithm” section). However, when the reads are too short and the coverage is low, there are many “tie cases” where multiple strains have the same *Vscore* as the reference strain. Table 1 shows the number of the tie cases out of the 2280 experiments for each case and also the median number of strains in the top ranking group based on *Vscore*. For example, when the reads have the length 75bp and coverage 5x, 903 out of 2280 strains have the top ranking group with at least 2 strains. The median number of strains in this group is 7. With the increase of the coverage, the tie cases drop significantly. When the coverage is 10x, the median number of strains in the top ranking group is 2. With the increase of the coverage, the tie cases reduce to 0 and the top 1 strain is always the correct reference. When the coverage is above 20x, VirStrain can return the correct strains without multiple hits. The change of read length does not significantly influence the performance when the depth is above 20x.

**Table 1:** The number of tie cases and the median number of best matches in all tie cases of VirStrain on 2280 ×20 simulated datasets

	75bp	100bp	150bp	250bp
5X	(903, 7)	(782, 6)	(602, 5)	(679, 5)
10X	(141, 2)	(109, 2)	(51, 2)	(65, 2)
20X	(0, 1)	(0, 1)	(0, 1)	(0, 1)
50X	(0, 1)	(0, 1)	(0, 1)	(0, 1)
100X	(0, 1)	(0, 1)	(0, 1)	(0, 1)

Each cell contains a tuple with the first number being the number of tie cases and the second number being the median number of strains in the top-ranking group. For example, “(903, 7)” in the combination 5x and 75bp means when the reads have the length 75bp and coverage 5x, 903 out of 2280 experiments return multiple strains with the same score. The median number of the returned strains is 7. For the single strain experiment, the ideal case is (0, 1), indicating that VirStrain ranks the correct strain as the top one

#### Impact of database size on VirStrain

To examine the relationship between the performance and the reference database size, we also repeated this experiment using databases with different sizes. The results are summarized in Supplementary File 1, Supplementary Table S1 [3, 30]. When the number of reference genomes decreases, the number of multiple hits slightly decreases for the same combination of read length and sequencing coverage.

#### Will the *k*-mer derived by VirStrain match non-viral genomes?

Because heterogeneous samples such as viral metagenomic data can contain reads from non-viruses, it is fair to ask whether VirStrain may construct false strains from non-viral reads. In order to evaluate this, we directly tested whether the *k*-mer derived by VirStrain can match commonly seen non-viral sequences, including those from human, bacteria, and bacteriophages. In addition, we tested whether there are *k*-mer matches between different viruses. The result is shown in Table 2. Most *k*-mer in the VirStrain database do not match the genomes of other species, indicating that VirStrain is not likely to mistaken other species as viral strains. Our experiments of applying VirStrain to real viral metagenomic data in the “VirStrain detects SARS-CoV-2 strains from real sequencing data” section further confirmed this.

**Table 2:** The number of *k*-mer matches between each type of virus and other two viruses, the human genome, bacteria, and bacteriophages

Virus		SARS-CoV-2	H1N1	HIV	Human	Bacteria	Phage
Name	# *k*-mer		(HA)	(Gag)	(GRCh38)		
SARS-CoV-2	34,754	-	0	0	1	0	0
H1N1 (HA)	687,818	0	-	0	13	12	0
HIV (Gag)	2,073,196	0	0	-	102	63	5

The human genome, 2770 complete representative bacterial genomes, and 3725 complete phage genomes are downloaded from NCBI RefSeq

### Detecting a reference strain from simulated reads

In this experiment, we compared VirStrain against Kraken2 [31], KrakenUniq [32], Pathoscope2 [11], Sigma [9], and Centrifuge [33] on detecting one reference strain from the input data. Although there are more taxonomic classification tools for sequence classification, other authors have shown that they cannot achieve satisfactory performance on strain-level composition [34]. Thus, we did not include those tools in our comparison.

For each tool, the reference database is constructed using RNA viral strains. As Sigma and Pathoscope2 are computationally expensive, we were not able to construct their database using all strains. To ensure a fair comparison using the same reference database, we built smaller, lower-resolution databases of 200 stains randomly selected from all strains of the three types of viruses.

For each virus, we randomly picked 100 strains/genes from the 200 reference sequences and simulated short reads from each. Thus, there are 300 datasets for three types of viruses. For each dataset, we used ART [29] to simulate 250 bp error-containing Illumina reads with depth of 100X, average insert size of 600 bp, and standard deviation of 150 bp. We identified strains from these simulated reads with VirStrain and five other tested programs and calculated the accuracy for each program.

**Fig. 3:**
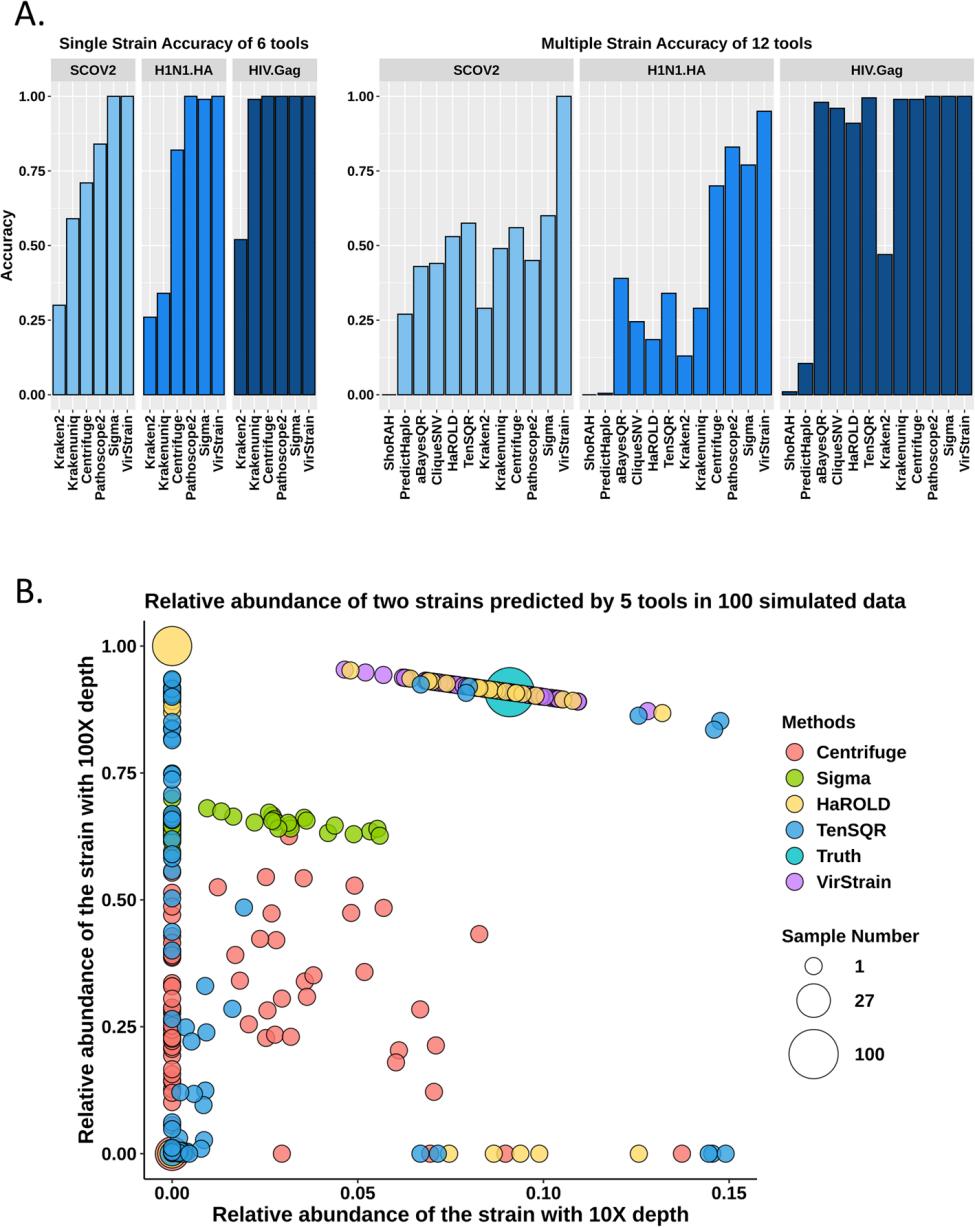
**A** The accuracy comparison of 12 tools. There are 100 sets of simulated reads for single-strain datasets and 100 for multi-strain datasets. For each set of multi-strain simulated reads, there are two strains with 100X and 10X coverage, respectively. **B** The bubble plot of the predicted abundance distributions for 100 simulated SARS-CoV-2 two-strain datasets. The center of each circle represents the relative abundance of the two strains output by one tool. When a tool produces the same abundance distribution on multiple datasets, we represent the identical output using a circle, whose size represents the number of those datasets. “Truth” refers to true relative abundance of the 2 strains in each dataset, which is calculated by normalizing the sequencing depth (100X and 10X). Its circle contains 100 datasets (samples). Many circles have centers with the *x*-coordinate being 0, meaning that these tools only output one strain

The performance comparison of different tools is shown in the left panel of Fig. 3A. Gag region of HIV shares relatively low similarity and thus it is easier to distinguish different reference genes. As a result, all tools have high accuracy. As H1N1.HA has very high similarity within the same clades or sub-clades, sequence classification tools that are not specifically designed for distinguishing highly similar genomes have low accuracy. We have similar observations for SARS-CoV-2 too. Across all the three viruses, VirStrain has consistently high accuracy. Tie cases were also checked for all tested tools. Only Centrifuge had tie cases (5/100 for SARS-CoV-2 and 9/100 for H1N1 (HA)) and no tie case was found in the output of all other tested tools.

In order to test the performance of VirStrain on all strains, we carried out a benchmark experiment for fast-running tools (see Supplementary File 1, Supplementary Section 1.2). The result shows that VirStrain is able to identify all strains in the database while other tools have lower accuracy (Supplementary File 1, Supplementary Figure S1).

VirStrain extracted about 30,000 *k*-mer out of roughly 300,000 *k*-mer from the input reference genomes. As shown in Fig. 3A, using all *k*-mer in Kraken2 does not render satisfactory accuracy. Based on this, it is noted that by selecting intelligently chosen unique combinations of *k*-mers centered around SNVs, strain distinguishment performs as well as if not better than the same program comparing all possible *k*-mers. Similarly, we observed decreased accuracy if we use all possible *k*-mer in VirStrain. Thus, using selected *k*-mer by the greedy covering algorithm is important to VirStrain.

#### Benchmark experiments on low coverage data

To assess the performance of other strain identification tools on datasets with low sequencing depth or shorter reads, we applied Krakenuniq, Centrifuge, Sigma, and Pathoscope2 on the same datasets used in Table 1 of the “Baseline performance of VirStrain” section. We did not evaluate Kraken2 due to its low accuracy on all tested viruses (Fig. 3A). To keep the same reference database configuration as the experiment in Table 1, all the tools used 4082 reference strain genomes (see Fig. 1B) except Sigma and Pathoscope2. Due to the high computational cost, we can only run Sigma and Pathoscope2 using a 200-strain reference database. The results were summarized in Supplementary File 1, Supplementary Table S2. All tested tools have poor performance on low coverage datasets except Sigma. Because Sigma was run on a much smaller reference database, the accuracy is expected to be higher. Nevertheless, Sigma took more than 2 weeks to analyze all datasets given 8 threads on an HPCC CentOS 6.8 node with 2.4Ghz 14-core Intel Xeon E5-2680v4 CPUs and 128 GB memory. On top of that, it has low accuracy in the mix-strain identification experiments (Fig. 3A).

### Detecting multiple strains from simulated data

Multi-strain infection is not rare for RNA viruses, especially the ones with high mutation rates. Usually, if one strain dominates the virus population, the minor strains tend to be missed. To mimic this situation, we constructed two-strain datasets that consist of a major strain (100x coverage) and a minor strain (10x coverage). Similar to “single-strain” datasets, we constructed 100 datasets of simulated reads for each type of virus. Each set contains simulated reads from two randomly selected reference sequences. The read simulation process is the same as the single strain experiment. As we know there are two strains, only the two most possible strains are kept for each tool. The accuracy is the ratio of correctly identified strains to the total number of the kept strains. Because haplotype reconstruction tools can be applied to assemble multiple strains, we also evaluated six haplotype reconstruction tools in this experiment. All constructed haplotypes by these tools are ranked according to their estimated frequency. For each predicted haplotype, we use MegaBLAST [35] to identify the closest reference strain (defined as *s*) in the database. If the reference strain *s* is the ground truth, we treat this haplotype as a correct identification. The performance comparison is shown in the right panel of Fig. 3A. Although the accuracy of VirStrain decreases a little for H1N1.HA (from 1.0 to 0.95) compared to the single-strain experiment, it maintains the accuracy of 1.00 for SARS-CoV-2. And it outperforms other tools by about 10% on H1N1(HA) and 38% on SARS-CoV-2.

Considering that haplotype reconstruction tools do not have the information of the known strains, one may wonder whether the accuracy-based metric is a fair evaluation of the haplotype reconstruction tools. We thus conducted in-depth evaluation of the constructed haplotypes by comparing their similarities with the ground truth. We focused on those predicted haplotypes whose closest strains *s* are not the ground truth. For each of these haplotypes, we first identified the reference strains that have higher similarity to the ground truth than *s*. Intuitively, a larger number of strains between *s* and the ground truth indicates worse accuracy of the haplotype reconstruction. We showed the numbers in Supplementary File 1, Supplementary Table S3 [35]. Then, we plotted the similarity distribution between those haplotypes and the ground truth in Supplementary File 1, Supplementary Figure S2. All tested tools tend to underestimate the number of strains (see Fig. 3B and Supplementary File 1, Supplementary Table S3), particularly the minor ones. ShoRAH [36] and PredictHaplo [37] have the worst performance and only reconstructed a few strains correctly. aBayesQR [38], CliqueSNV [24], and TenSQR [39] have relatively better performance than other tools. The constructed haplotypes by CliqueSNV have the highest similarity with the ground truth strains. However, it missed many strains, particularly the minor ones in the input data. Overall, the outputs of the haplotype reconstruction tools cannot reflect the true strain composition.

#### Recombinant strains

One related question is whether VirStrain is able to distinguish recombinant strains from their parent strains. VirStrain is able to detect recombinant strains like other strains when it is included in the reference database. We applied VirStrain to identify recombinant strains from 2 simulated mix-strain datasets (see Supplementary File 1, Supplementary Table S4) [40, 41]. The results show that VirStrain can identify both the recombinant strain and the strains in its parent genotypes/subtypes. However, VirStrain is not designed to distinguish the recombinant strain from its parent strains because the recombinant strain may not possess enough SNVs.

#### Relative abundance computation

For identified strains, VirStrain also outputs its sequencing coverage, which can be used to compute relative abundance for multi-strain infection. As Fig. 3A shows, the accuracy of ShoRAH, PredictHaplo, CliqueSNV, aBayesQR, Kraken2, KrakenUniq, and Pathoscope2 on the SARS-CoV-2 multiple-strain data sets is lower than 0.5. Thus, we did not include them in the comparison. Sigma, Centrifuge, HaROLD [42], and TenSQR were able to return the strains’ abundances in the outputs. Therefore, it is convenient to calculate the relative abundance for each strain.

Figure 3B shows that the relative abundance estimated by VirStrain is closer to the ground truth than others. Sigma, Centrifuge, HaROLD, and TenSQR failed to detect the minor strain in many datasets. Thus, many data points are aligned with *x*-value 0.00. In addition, they have more variations about the relative abundance computation for different datasets even though the ground truth keeps the same (100x vs 10x).

### VirStrain detects the closest relative for novel strains

When a strain is not present in the reference database, VirStrain will output its closest relative in the database. Here, we define the closest relative as the strain in the database that is most similar to the query strain identified by MegaBLAST [35]. In order to test VirStrain on returning the closest relative for novel strains, we created multiple simulated read sets from mutant strain genomes.

In order to test the ability of different tools on detecting the closest relative, we need to reconstruct our reference genome set by choosing *only the sequences that can be correctly identified by all tools*.

Thus, we used 53 SARS-CoV-2 genome sequences that can be identified correctly by all tools in the single-strain experiment. Then, we used simuG program [43] to simulate random point mutations to each of these genomes. According to Dorp et al. [44], the average number of mutations in the SARS-CoV-2 strains is 9.6. Thus, we simulated mutant genomes with 5, 7, 9, 11, and 13 random point mutations from the raw genome sequences and marked these newly obtained genomes as M5, M7, M9, M11, and M13. In total, there are 265 (53*5) mutant genomes and 53 raw (i.e., reference) genomes. Then, we simulated short reads from these mutant and raw genomes using the same parameters as other experiments. Thus, we have 318 (265 mutant and 53 raw) datasets as inputs. For each dataset, as it only contains reads simulated from one strain, we thus only keep the top 1 output by different tools.

**Fig. 4:**
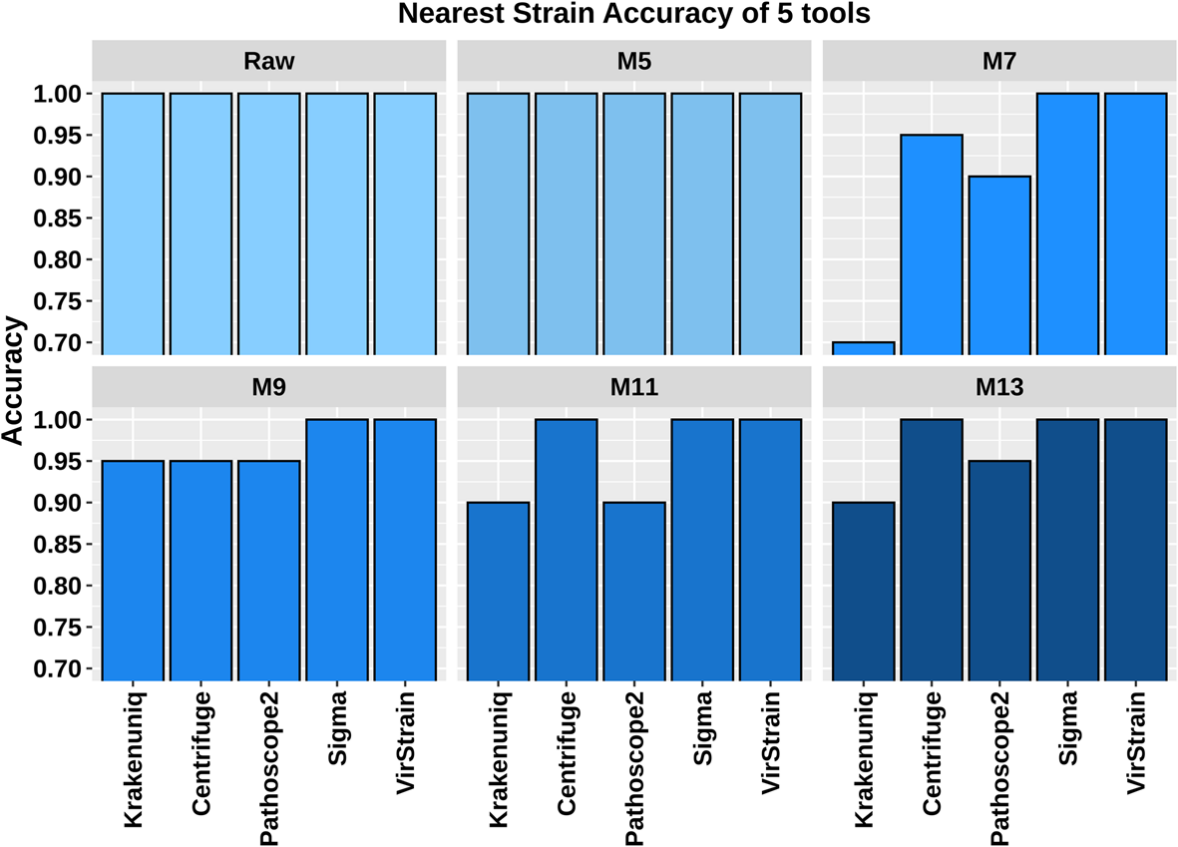
The accuracy comparison of 5 tools on detecting the closest relative in 318 simulated datasets. The 53 strains used in this experiment can be correctly identified by all tools in Fig. 3. “Raw” means the dataset from the reference genomes and M5, M7, M9, M11, and M13 represent datasets simulated from mutant strains. There are a total of 53 data sets for each group and each dataset contains one strain

Figure 4 shows that VirStrain and Sigma are able to find the correct closest relatives in all data sets, while the other 3 tools failed to output the correct strains in some cases. This is consistent with the experimental results of Sigma [9], which tested this function on multiple datasets.

This experiment demonstrated that the performance of our tool is as good as the mapping-based tool Sigma in identifying the closest relative. It is noteworthy that Fig. 4 looks better than Fig. 3 for several tested tools because this experiment only used 53 strains that are correctly identified by all tools in the single-strain experiment.

To further test the robustness of VirStrain, we applied VirStrain to detect the closest relative in a larger simulated dataset (see Supplementary File 1, Supplementary Section 1.6). The result shows that VirStrain can still identify correct closest relatives in all the 600 simulated datasets (Supplementary File 1, Supplementary Figure S3).

### Running time comparison

To evaluate the computational efficiency of VirStrain, we compared the running time of the tested tools on the simulated data and recorded the result in Table 3. One real metagenomic sequencing data (SRR10971381) is also used to compare the computational efficiency due to its large data size. The reference genome of the strain (MN908947) in this dataset (SRR10971381) can be found in the database of all tested tools, so it is a fair test strain. VirStrain has similar running time to Centrifuge and KrakenUniq but runs significantly faster than Pathoscope2 and Sigma. All the experiments were tested on an HPCC CentOS 6.8 node with 2.4Ghz 14-core Intel Xeon E5-2680v4 CPUs and 128 GB memory. We used 8 threads for all tools. We also evaluated the running time of haplotype reconstruction tools in Supplementary File 1, Supplementary Section 1.12 and found all tested tools were more computationally expensive than VirStrain. These results indicate that VirStrain achieved much higher accuracy than those computationally efficient tools such as KrakenUniq and Centrifuge while maintaining comparable speed. It also outperforms those mapping-based tools like Sigma and Pathoscope2 on both accuracy and speed.

**Table 3:** Running time of five tested tools on simulated and real data

Data sets	VirStrain	KrakenUniq	Centrifuge	Pathoscope2	Sigma
Sim_single_strain (6 MB)	16s	8s	6s	110s	255s
Sim_multiple_strain (6.6 MB)	23s	9s	8s	140s	268s
SRR10971381 (19.5 GB)	215s (Y)	254s (N)	290s (N)	1721s (Y)	>15h (-)

Sim_single_strain and Sim_multiple_strain represent simulated single-strain and multiple-strain datasets, respectively. For real data, the identification result is represented by Y and N, where Y means correct identification and N means wrong identification. Sigma does not have the identification result due to its long running time

**Table 4:** The VirStrain identification result of 32 real sequencing datasets

BioSample	Sequencing	Blast	VirStrain	Data	Running	Region	Region	Genome
accession number	platform	result	result	size	time	of sample	of clusters	in the DB
SAMN13922059	Illumina MiniSeq	MT470175.1	MT470175.1	19.5 GB	215s	Wuhan, China	Hangzhou, China	
SAMN14652901	Illumina MiSeq	MT582484.1	MT582484.1	1.08 GB	34s	Heinsberg, Germany	Dusseldorf, Germany	
SAMN15062833	NextSeq 550	MT327745.1	MT327745.1	3.4 GB	39s	Turkey	Turkey	
SAMN17816674	Illumina MiSeq	MT680219.1 (>1 hit)	MT680219.1	94 MB	24s	New Mexico, USA	Virginia, USA	
SAMN15941290	Ion Torrent S5	MT576531.1	MT576531.1	625 MB	19s	Gujarat, INDIA	Gujarat, INDIA	
SAMEA7098097	Illumina MiSeq	MT520283.1	MT520283.1	1.2 GB	20s	Stockholm, Sweden	Massachusetts, USA	
SAMN15916668	NextSeq 500	MT745701.1	MT745701.1	309 MB	14s	Victoria, Australia	Victoria, Australia	
**SAMN14560168**	Illumina iSeq 100	MT470175.1	MT470175.1	2.8 GB	47s	Cambodia	Hangzhou, China	
**SAMN14643484**	Illumina MiSeq	MT159710.2	MT159710.2	1.1 GB	31s	Israel	DP cruise ship	
SAMN17799471	Illumina MiSeq	MT506654.1	MT506654.1	72 MB	29s	Ohio, USA	Michigan, USA	
SAMN14652902	Illumina MiSeq	MT582484.1	MT582484.1	1.71 GB	44s	Heinsberg, Germany	Dusseldorf, Germany	N
SAMN14652906	Illumina MiSeq	MT582496.1	MT582496.1	1.16 GB	34s	Heinsberg, Germany	Heinsberg, Germany	N
SAMN17516427	Illumina NovaSeq 6000	MT558692.1 (>1 hit)	MT558692.1	618 MB	31s	Rhode Island, USA	Virginia, USA	
SAMN17516424	Illumina NovaSeq 6000	MT558692.1	MT558692.1	768 MB	31s	Rhode Island, USA	Virginia, USA	N
SAMN17516418	Illumina NovaSeq 6000	MT558692.1 (>1 hit)	MT558692.1	668 MB	28s	Rhode Island, USA	Virginia, USA	
SAMN17816685	Illumina MiSeq	multiple_hits (>2 hits)	MT585085.1	774 MB	32s	New Mexico, USA	USA	
SAMN17911680	Illumina MiniSeq	multiple_hits (>2 hits)	MT576531.1	110 MB	20s	Chattogram, Bangladesh	Ahmedabad, INDIA	
SAMN17855793	Illumina MiSeq	MT374114.1 (TaiWan)	MT371002.1	108 MB	20s	California, USA	New York, USA	N
SAMN17816704	Illumina MiSeq	MT293195.1	MT680219.1 (blast_rank =2)	114 MB	33s	New Mexico, USA	Virginia, USA	
SAMN17814567	NextSeq 550	MT344948.1 (v_rank =3)	MT750348.1	1.21 GB	36s	USA	California, USA	
SAMN17855792	Illumina MiSeq	MT259281.1	MT370994.1	108 MB	20s	California, USA	New York, USA	N
SAMN17486870	Illumina MiSeq	MT263459.1	MT460132.1	91 MB	21s	USA	California, USA	N
SAMN17486876	Illumina MiSeq	MT263406.1	MT536186.1	64 MB	29s	USA	New Orleans, USA	N
SAMN17486898	Illumina MiSeq	MT192765.1	MT750383.1	85 MB	25s	USA	California, USA	N

### VirStrain detects SARS-CoV-2 strains from real sequencing data

#### Apply VirStrain to trace the infection location

To evaluate the performance of VirStrain in SARS-CoV-2 strain identification, we conducted experiments on 32 real sequencing datasets (see Table 4), which were sampled from patients of different geographical regions. The samples were sequenced using different platforms such as Illumina, BGI-Seq, and Ion Torrent and may not have complete assemblies available. There are 7 viral metagenomic samples. Out of the 32 samples, 4 samples have their SARS-CoV-2 strains present in the VirStrain database. Sixteen of them have available complete genomes but they are not in our reference database (the samples marked with red in Table 4). For the remaining samples, not every one can be assembled into complete SARS-CoV-2 genomes. We applied 3 popular assembly tools and found none of these tools can assemble the short reads into complete genomes for four datasets (see Supplementary File 1, Supplementary Section 1.7) [45, 46]. Even so, VirStrain can still be applied to identify the closest strain in the reference database and uses the metadata to provide possible infection locations. The results are shown in Table 4.

**Table 4:** The VirStrain identification result of 32 real sequencing datasets *(Continued)*

BioSample	Sequencing	Blast	VirStrain	Data	Running	Region	Region	Genome
accession number	platform	result	result	size	time	of sample	of clusters	in the DB
SAMN15144727	BGISEQ-500	Unknown	MT568634.1	32 MB	13s	Guangzhou, China	Guangzhou, China	N
SAMN15637956	Illumina HiSeq 4000	Unknown	MT066175.1	6.8 GB	114s	China	Guangzhou, China	N
SAMN16058334	NextSeq 500	Unknown	MT633030.1	8.2 GB	73s	Washington, USA	Washington, USA	N
SAMN16068353	NextSeq 500	Unknown	MT345882.1	320 MB	33s	Nevada, USA	Washington, USA	N
SAMN16068354	NextSeq 500	Unknown	MT641532.1	612 MB	16s	Nevada, USA	Washington, USA	N
SAMN15678404	NextSeq 500	Unknown	MT632835.1	974 MB	29s	Washington, USA	Washington, USA	N
SAMN15678405	NextSeq 500	Unknown	MT375468.1	539 MB	27s	Washington, USA	Washington, USA	N
SAMN14668182	Ion Torrent S5	Unknown	MT704132.1	24 MB	13s	New York, USA	Maryland, USA	N

“Unknown” in the column “Blast result” means that the complete genome of that dataset is not available. “Region of clusters” is the output of VirStrain based on the metadata associated with the reference strains in each cluster. For clusters containing more than one reference strain, we use the majority vote to get the geographical region information. “Genome in the DB” represents whether the complete genome of that dataset can be found in the reference database of VirStrain, yes (Y) or no (N), and the red character means these samples have complete genomes. “DP cruise ship” refers to the Diamond Princess cruise ship. “v_rank” represents the ranking of the strain in the output of VirStrain. “blast_rank” represents the ranking of the strain in the output of Blast

By comparing the metadata of the output strain by VirStrain and the known information associated with each sample, we can conclude that the derived and known geographical information is generally consistent for all datasets. For most cases where the complete genomes are available, the strains returned by VirStrain are the same as the output of MegaBLAST. There are four cases where VirStrain output different results from MegaBLAST. Of the four cases, MegaBLAST output multiple hits for two. For the other two, the strain identified by VirStrain is very close to MegaBLAST. As VirStrain uses short reads as input, this indicates that its accuracy is comparable to highly accurate alignment tools that take genomes as input.

The first sample SAMN13922059 is actually from a patient in Wuhan, China, whose sample was used to generate the first reference genome of SARS-CoV-2 [47]. In the output of VirStrain, this first reference genome is located in a cluster with other 47 SARS-CoV-2 strains, which all belong to clade 19A defined by nextstrain. In this cluster, there are two main geographical locations: Wuhan and Hangzhou, China. As Hangzhou’s cases are slightly more than Wuhan, we used Hangzhou in column “Region of clusters”. This is one current limitations of VirStrain. These 48 strains cannot be divided into single-strain clusters.

There are 2 very interesting samples in Table 4: SAMN14560168 and SAMN14643484 (bold font). SAMN14560168 is from the first COVID-19 patient of Cambodia, who had been to China before being admitted to the hospital. The identification result of VirStrain shows that its closest relative is MT470175.1, which is from China. Thus, the result indicates that this Cambodia patient could be infected in China, which is consistent with this patient’s travel history. Another interesting case, SAMN14643484, is from Israel and its closest relative identified by VirStrain is from the Diamond Princess cruise ship. According to the sample information at NCBI, this patent was indeed a passenger of the cruise ship and got infected by SARS-CoV-2 there.

These results show that VirStrain is able to identify SARS-CoV-2 strains from real sequencing data with or without assembled genomes. In addition, VirStrain also provides information that can be very useful for tracking the virus spread.

#### Apply VirStrain to identify co-infection of SARS-CoV-2 strains

**Fig. 5:**
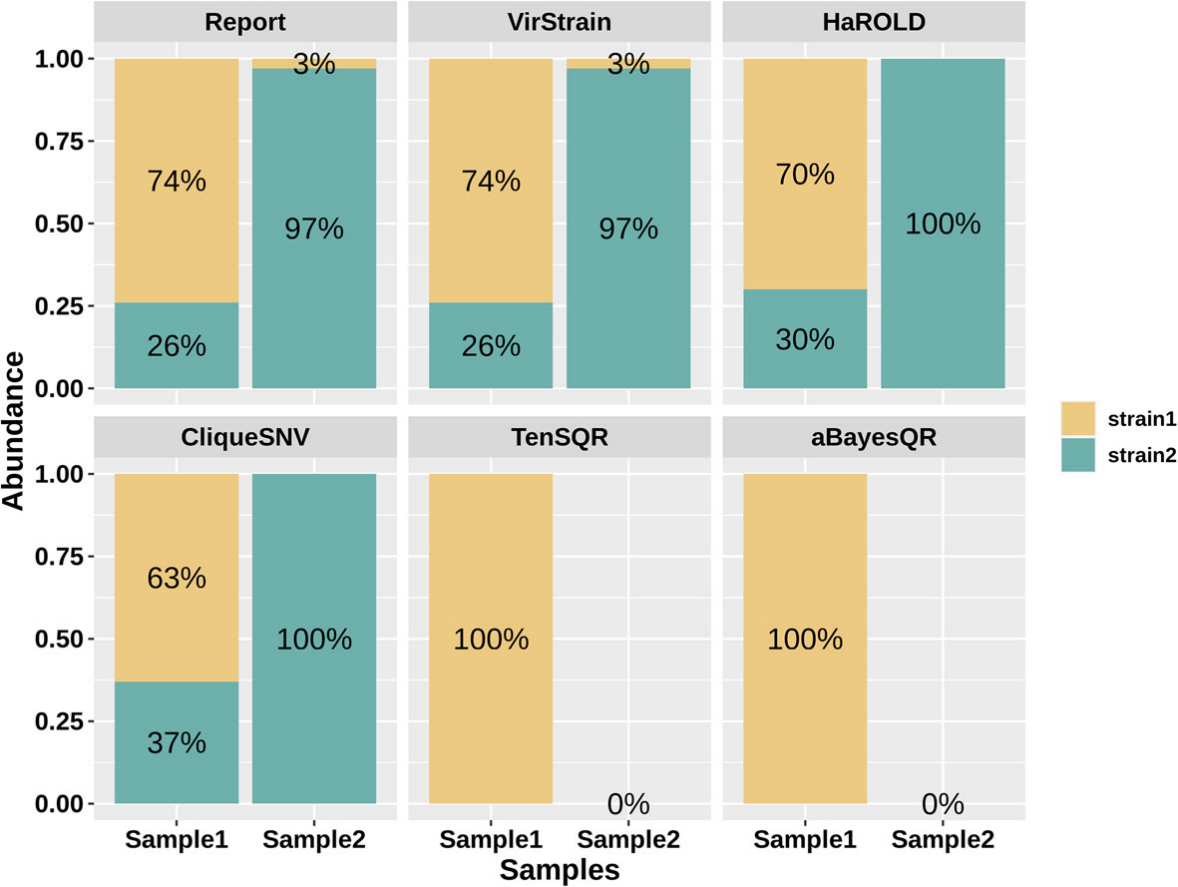
The comparison between the reported abundance in the original study (referred to as “report”) and the predicted by 5 tools. Strain1 belongs to clade 20C and strain2 belongs to clade 20B defined by nextstrain [3]. According to the original study, there are only 8 mutations between strain1 and strain2. TenSQR failed to analyze “Sample2” due to memory usage, so the abundance is “0%.” Similarly, aBayesQR was terminated after analyzing Sample2 for more than 7 days using 8 threads, so the abundance is also “0%”

A recent study [48] reported a case where one patient was infected by 2 different SARS-CoV-2 strains simultaneously. According to the authors, two samples (Sample1 and Sample2) were obtained from the same patient 8 days apart and both samples were found to contain two highly similar SARS-CoV-2 strains from different clades. In addition, they also found a change in the dominant strain between Sample1 and Sample2. Thus, we applied VirStrain, HaROLD, CliqueSNV, TenSQR, and aBayesQR to these 2 samples (SRR14142137 and SRR14142136) to check whether they were able to identify the co-infection of SARS-CoV-2. In Fig. 5, we compared the results obtained from the original study and 5 tools. As shown in Fig. 5, VirStrain is the only tool that identifies two strains with the same clades as the original study in two samples and is consistent with the original study in terms of abundance prediction. This experiment shows that VirStrain can provide useful insights into strain co-infection, even those that are highly similar and have low abundance.

### VirStrain identifies 5 strains from HIV mock data

In this experiment, we applied VirStrain to a mock dataset (SRR961514) containing real sequencing data from five HIV strains. The authors mixed five HIV strains (JRCSF, 89.6, NL43, YU2, and HXB2) and conducted Illumina sequencing [49]. Using the reads as input, VirStrain can detect 5 strains from its reference database and predict their sequencing depth. Based on the predicted sequencing depth, we calculated the relative abundance by normalizing the depth of each identified strain.

**Fig. 6:**
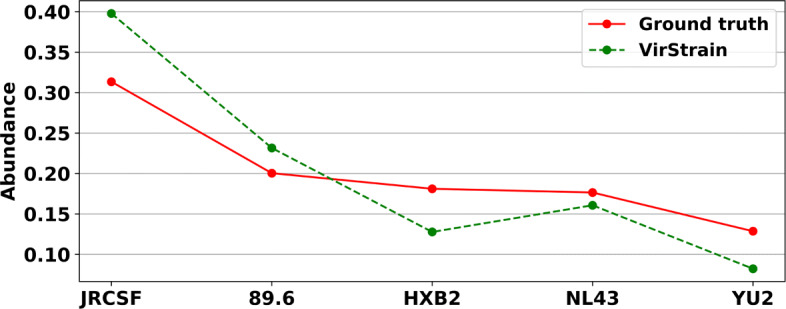
Abundance comparison of HIV mock data between the ground truth and VirStrain. The true average abundances sorted in descending order are 31.35%, 20.04%, 18.11%, 17.65%, and 12.86%

To compare the predicted abundance with the ground truth, we applied the chi-square test and got the *p*-value 0.9998, which indicates that the distribution of the predicted abundance by VirStrain is not statistically different from the ground truth (Fig. 6). This experiment demonstrates the ability of VirStrain in identifying multiple strains in one sample.

### VirStrain identifies strains of DNA viruses from mix-strain sequencing data

Because there are a large number of sequenced strains of high similarity for RNA viruses, we mainly focused on evaluating VirStrain on RNA viruses. But VirStrain can also be applied to DNA viruses as long as a quality multiple sequence alignment can be produced for the reference strain genomes. Mixed strain infections are also possible for DNA viruses, such as hepatitis B virus (HBV) and human cytomegalovirus (HCMV). To further assess the performance of VirStrain on the mixed strain identification of DNA viruses, we applied VirStrain to 12 real sequencing datasets, which consist of 2 HBV samples and 10 HCMV samples with known strain composition.

The two samples for HBV are from the same study (PRJEB31886) and both contain two known strains of HBV [50]. To test the performance of VirStrain on these datasets, we downloaded the complete genomes of 9356 HBV strains from NCBI and then applied VirStrain to build the reference database. We also tested the performance of two other efficient tools, Krakenuniq and Centrifuge, and 4 haplotype reconstruction tools on the same datasets. The result shows that only VirStrain can successfully identify all strains present in the samples (Table 5). Centrifuge can identify the dominant strains but miss the low abundance strains. The performance of the haplotype reconstruction tools can be found in Supplementary File 1, Supplementary Table S6. Because the strains’ similarity is 89.97%, much lower than RNA viruses, all tested tools can output haplotypes with the highest similarity to the ground truth strains. But the constructed haplotypes are not identical to the ground truth. And none of the tools is consistently better than others on the two datasets. For example, the haplotypes output by aBayesRQ have the highest similarity (97.7% and 99.8%) to the true strains on one of the sample. But HaROLD and CliqueSNV generated more accurate haplotypes on the other sample.

**Table 5:** Performance of the three tools on two HBV mix-strain datasets

Sample name	Actual strains present in the sample	VirStrain		Centrifuge		Krakenuniq	
		Strains detected	Predicted abundance	Strains detected	Predicted abundance	Strains detected	Predicted abundance
ERR3253398	MK720631.1	Y	89%	Y	12%	N	-
	MK720628.1	Y	11%	N	-	N	-
ERR3253399	MK720631.1	Y	80%	Y	14%	N	-
	MK720628.1	Y	20%	N	-	N	-

“-” in the table indicates that the strain is not identified and thus the abundance is unknown

We then evaluated VirStrain using 10 HCMV samples, which are from a comprehensive benchmark study for tools on strain-resolved analysis [51]. According to the authors, these 10 lab-generated strain mixtures were generated from 3 HCMV strains (TB40/E, AD169, and Merlin), at different mixing ratios. For example, the sample “TA-1-1” in Table 6 means it was generated from HCMV strains TB40/E and AD169 (designated as “TA”), at a mixing ratio of 1:1. Then, we downloaded the complete genomes of 332 HCMV strains from NCBI and constructed the database using 328 strains with the parameter “-s 0.4”. Four strains are not included due to the low quality such as frameshift errors in many genes. Six samples contain two strains and thus we also tested HaROLD on them because HaROLD was adopted by another study to reconstruct HCMV strains in clinical samples [52]. The result is shown in Table 6. On these 10 samples, VirStrain achieves 100% accuracy. For two hard cases (“TA-1-50” and “TM-1-50”), VirStrain is able to successfully identify both the dominant and low abundance strains. However, the accuracy of HaROLD is only 50%. For the three samples of “TM”, HaROLD is only able to successfully reconstruct the genome sequence of Merlin, which is consistent with our previous observation that HaROLD tends to underestimate the number of strains.

**Table 6:** Performance of VirStrain and HaROLD on 10 HCMV lab-generated benchmark datasets

ID	Sample name	Actual strain present in the sample	VirStrain		HaROLD	
			Strains detected	Predicted abundance	Strains detected	Predicted abundance
1	TA-0-1	AD169	Y	100%	-	-
2	TA-1-0	TB40/E	Y	100%	-	-
3	TA-1-1	TB40/E	Y	83%	Y	79%
		AD169	Y	17%	Y	21%
4	TA-1-10	TB40/E	Y	56%	Y	30%
		AD169	Y	44%	Y	70%
5	TA-1-50	TB40/E	Y	5%	Y	5%
		AD169	Y	95%	Y	95%
6	TM-0-1	Merlin	Y	100%	-	-
7	TM-1-0	TB40/E	Y	100%	-	-
8	TM-1-1	TB40/E	Y	66%	N	0%
		Merlin	Y	34%	Y	100%
9	TM-1-10	TB40/E	Y	16%	N	0%
		Merlin	Y	84%	Y	100%
10	TM-1-50	TB40/E	Y	5%	N	0%
		Merlin	Y	95%	Y	100%

“-” in the table indicates that HaROLD is not tested on these datasets because they contain only one strain. The three strains are TB40/E (T), AD169 (A), and Merlin (M). Each sample name starts with the acronyms of the two composite strains, followed by the strain ratio

### Application of VirStrain to a cohort with mixed HCMV infections

In this experiment, we applied VirStrain to samples (PRJNA605798) collected from five HIV-infected Kenyan mothers and their infants between 1993 and 1998 [53]. These samples are sequenced at different time points from the mother’s breast milk (BM), cervical (CV), and the infant’s blood spots (BS). In a recent study [52], the authors used HaROLD to reconstruct and analyze the HCMV strains in this batch of samples. Here, we take a similar approach to the original study to show the utility of VirStrain in identifying mixed strain infections. It is worth noting that “family” in this experiment represents all samples of a mother and her infant, so the subsequent analysis contains a total of 5 families.

**Fig. 7:**
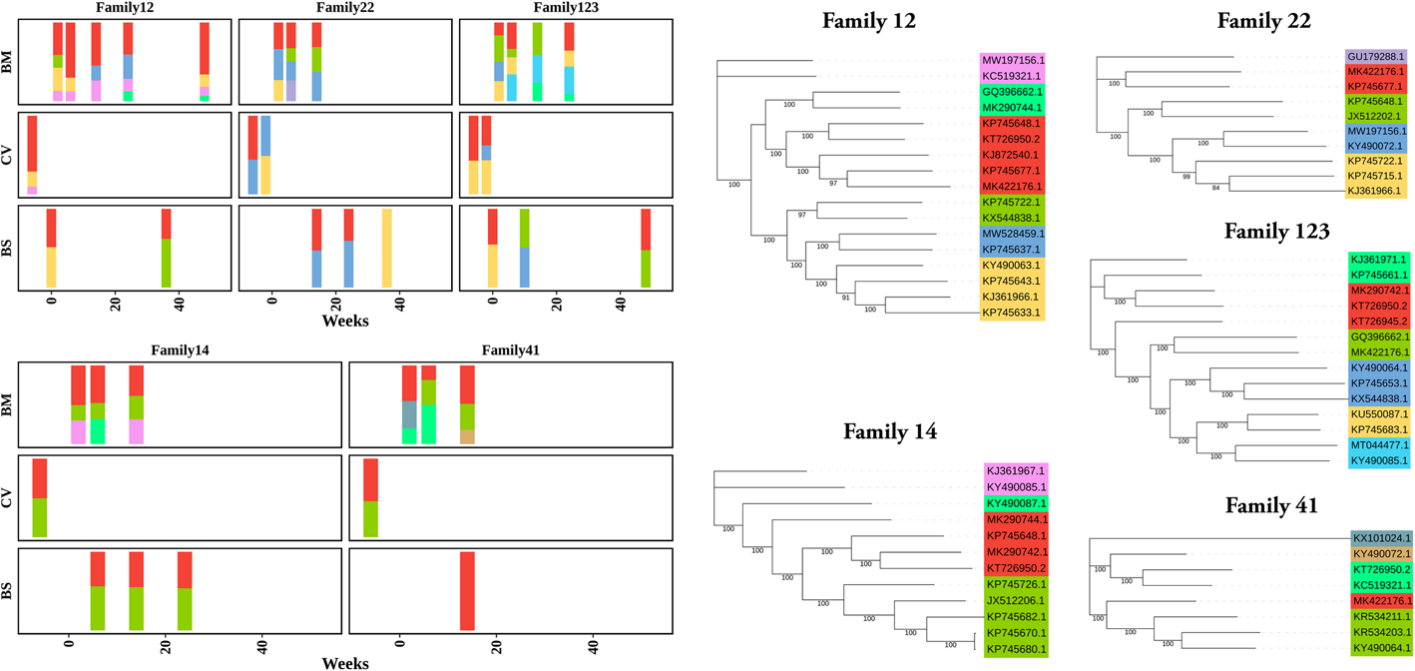
Abundance of strains within each sample for breast milk (BM), cervix (CV), and blood spots (BS), and the maximum-likelihood phylogenetic tree for strain clusters (genotypes) of each family. Each color in the left panel is associated with the genotypes’ color on the right side. For all families, we use the same set of colors to represent different genotypes. However, for different families, the same colors are not directly related and do not represent the same genotype. The value shown in the tree represents the bootstrap value. The visualization of phylogenetic tree is generated with iTOL [56]

Firstly, we applied VirStrain to identify HCMV strains in these longitudinal samples with the same reference database as mentioned in the “nameref7dnamixdata” section. For the strains identified by VirStrain, we used Mafft v7.455 [54] to align their genomes and constructed maximum-likelihood trees of the strains from each family using FastTree v2.1.11 [55]. Similar to the original study, the strains were then grouped into clusters and the pairwise evolutionary distance between each strain pair of a cluster was less than 0.017. Same as the original study, the evolution distance here refers to the sum of the distances between the strains and their latest common ancestor on the evolutionary tree. As a result, 26 clusters were generated and we considered these clusters as genotypes. The different strain clusters (genotypes) were represented by different colors in Fig. 7. In Fig. 7, we plot the abundance of each genotype within a sample over time to visualize the strain composition relationship between maternal and infant genotypes.

As shown in Fig. 7, there are multiple genotypes in the breast milk of all five mothers and the relative abundances change over time. Besides, we can also have some interesting findings regarding the mother-to-child transmission of HCMV strains. For example, the infants from families 12 and 123 were initially infected with genotypes found in the cervix and then re-infected with genotypes found only in breast milk. Same as the original study, we also found a distinct genotype (marked in purple) in mother 22’s breast milk in the first 6 weeks, which disappeared in subsequent samples. However, the original study based on HaROLD analysis indicated that most samples from cervical and infant blood spots contained only a single genotype, but our analysis showed that most of these samples contained two genotypes. This may be the result of HaROLD’s tendency to underestimate the number of strains. More interestingly, we find that the genotypes found in the breast milk all contain strains (MK422176.1, MK290742.1, etc.) isolated from the breast milk of HIV-infected mothers in Zambia [57], and similarly, some of the strains (KJ361966.1, KR534203.1, etc.) identified in cervical are from the amniotic fluid. These results suggest that VirStrain can provide more comprehensive views for the analysis of mixed strain infections.

## Discussion

Our large-scale benchmark experiments against several other popular strain-level analysis tools and haplotype reconstruction tools demonstrated the high accuracy of VirStrain on detecting reference strains from short reads. But there are still cases where VirStrain cannot return the exact strain. One limitation of the current method is the ambiguity of detecting low abundance viral strains from very short reads (depth <10x, read length <100bp). As mentioned in the “Baseline performance of VirStrain” sections, there could be multiple best matches when the depth is smaller than 10X. As not all SNV sites can be covered by the reads, strains of high similarity and with a large number of shared SNVs can form a tie case with the same score. With coverage bigger than 10x, the tie cases become very rare and the top 1 strain identified by VirStrain is the correct strain in the sample. This limitation caused by low coverage and high similarity is also observed in other tested tools. Instead of outputting a wrong strain, VirStrain outputs all with the correct strain being one of them, which can inform the users of this ambiguity. It is our future work to design more accurate algorithms for addressing this limitation.

In the case of detecting multiple strains in one sample, there is a tradeoff between the resolution and accuracy. Specifically, if there are multiple strains sharing a large number of SNV sites, they will be clustered in the list ranked by *Vscore*, which can pose false positive detection when one of the strain exists in the underlying sample. Thus, we do not reuse the SNV sites so that the output strains are representative ones in a sample rather than near duplicate ones. Essentially, the iterative search procedure poses a constraint on the number of different SNVs between strains in the same sample. Strains with too few differences will be missed by VirStrain. In order to provide the guidance on the number of expected different SNVs, we tested a hard case for our method. The input data contains reads from three strains, with one being the major one (100x) and other two being minor ones (10x). In addition, two of the three strains are highly similar with less than 10 different SNV while the rest one has more than 3 different SNVs. The result can be found in Supplementary File 1, Supplementary Section 1.11. It shows that VirStrain may miss the low abundance strain that differ by less than 10 SNV sites for SARS-CoV-2 in the multi-strain infection cases.

Currently, we derive *k*-mer from aligned reference genomes. Thus, VirStrain is not designed for bacterial strain identification because it is hard and computationally expensive to obtain the high-quality multiple sequence alignment of bacterial strain genomes. We noticed some alignment errors especially at sites with consecutive insertions or deletions. As a result, we tend to exclude columns with many indels, which may lead to clusters containing multiple genomes in the end. Ideally, we want to derive optimal *k*-mer sets for reference genomes without relying on alignment programs, which is our future work.

It also should be noted that the completeness and bias of the genomes in the database will play a major role in the performance of VirStrain. For example, low-quality genomes may lead to poor quality of multiple sequence alignment, which may affect the accuracy of VirStrain. VirStrain does not automatically detect the contamination or bias of the database. But VirStrain allows the users to build the reference database using their own reference genomes. Thus, data pre-processing can be conducted to mitigate the bias or contamination.

## Conclusions

In this work, we implemented a strain identification tool for short reads. We designed a greedy covering algorithm to divide reference genomes into multiple clusters so that the genomes in each cluster possess unique set of *k*-mer.

VirStrain shows higher accuracy than other tested tools across all benchmark datasets with different complexity. VirStrain can be applied to identify strains from low-quality sequencing data, which is the hard case for assembly tools (see Supplementary File 1, Supplementary Table S5). VirStrain has high accuracy in detecting multi-strain infection cases. We demonstrated this by using VirStrain on both simulated and real sequencing data on different types of viruses including SARS-CoV-2, HIV, H1N1, HBV, and HCMV.

## Material and methods

In this work, we developed a tool, VirStrain, which can quickly identify one or multiple reference strains closest to those in short-read sequencing data. It achieves a better tradeoff between speed and resolution by deriving unique *k*-mer combinations that can distinguish highly similar strains.

VirStrain conducts strain identification using short reads as input and does not rely on sequence assembly, making it more amenable to cases where full virus genome cannot be assembled. The output of VirStrain contains the most possible strain (the strain that best matches the SNVs found in the sample set) identified in the data and the detailed read coverage of its single nucleotide variation (SNV) sites in an interactive HTML format (Supplementary File 1, Supplementary Figure S5).

**Fig. 8:**
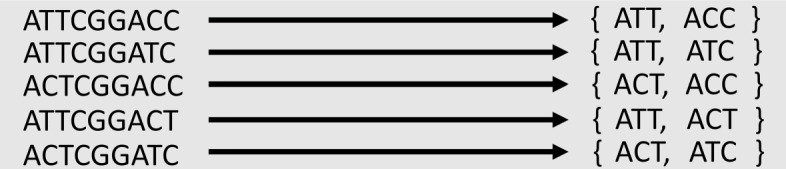
Use *k*-mer sets to distinguish five sequences of high sequence similarity. Each sequence has a unique *k*-mer combination

Highly similar reference genomes may not possess genome-specific *k*-mers. But they can possess genome-specific *k*-mer set, where the component *k*-mer can be utilized together to distinguish different reference genomes. Figure 8 shows a toy example of using *k*-mer sets to distinguish five sequences when there are no genome-specific *k*-mer. In order to find such *k*-mer set, we develop a greedy covering algorithm to identify unique combinations of SNV sites from aligned virus reference genomes. Then, *k*-mer will be extracted from the SNV sites and construct *k*-mer set for underlying genomes.

### Step 1: identify unique set of SNVs from reference genomes

The input to this algorithm is an MSA of the reference genomes. It is noteworthy that generating MSA for thousands to tens of thousands of genomes can be slow. But when the reference genomes share high sequence similarity (such as for SARS-CoV-2), the MSA can be produced using more efficient programs, such as the one provided by Mafft at its website [54].

Given the MSA $\mathcal {M}$, the program will exclude all the sites where no SNV is observed. Instead, the algorithm favors variations from conserved sites, which indicate features that are specific to one or a small number of genomes. Thus, given $\mathcal {M}$, we compute the Shannon entropy $\mathcal {H}$ for each column.


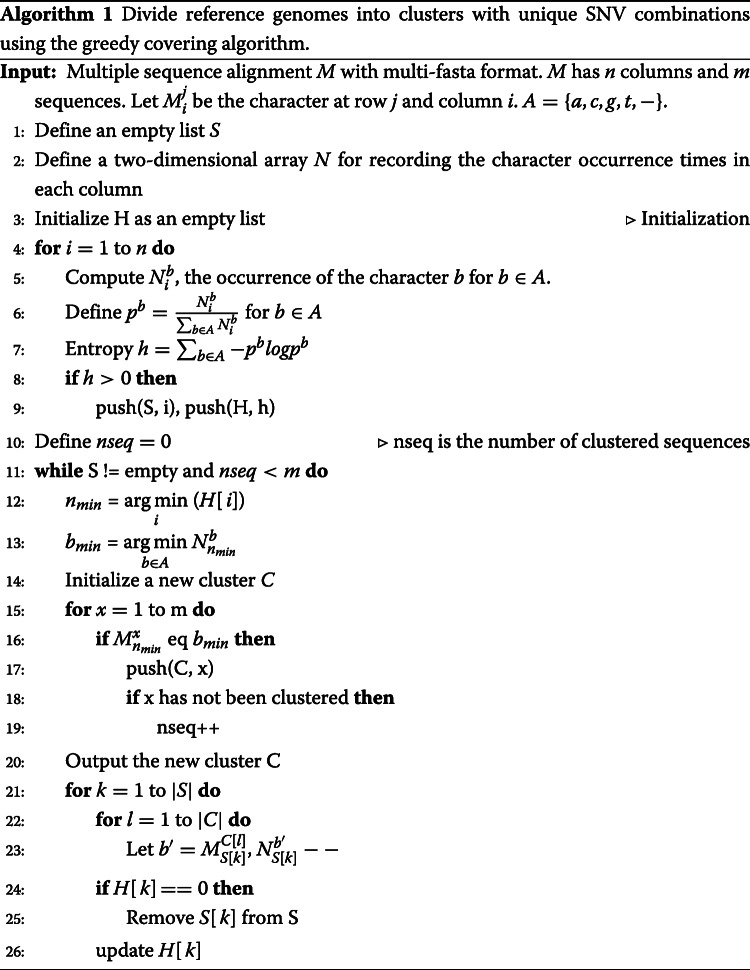


**Fig. 9:**
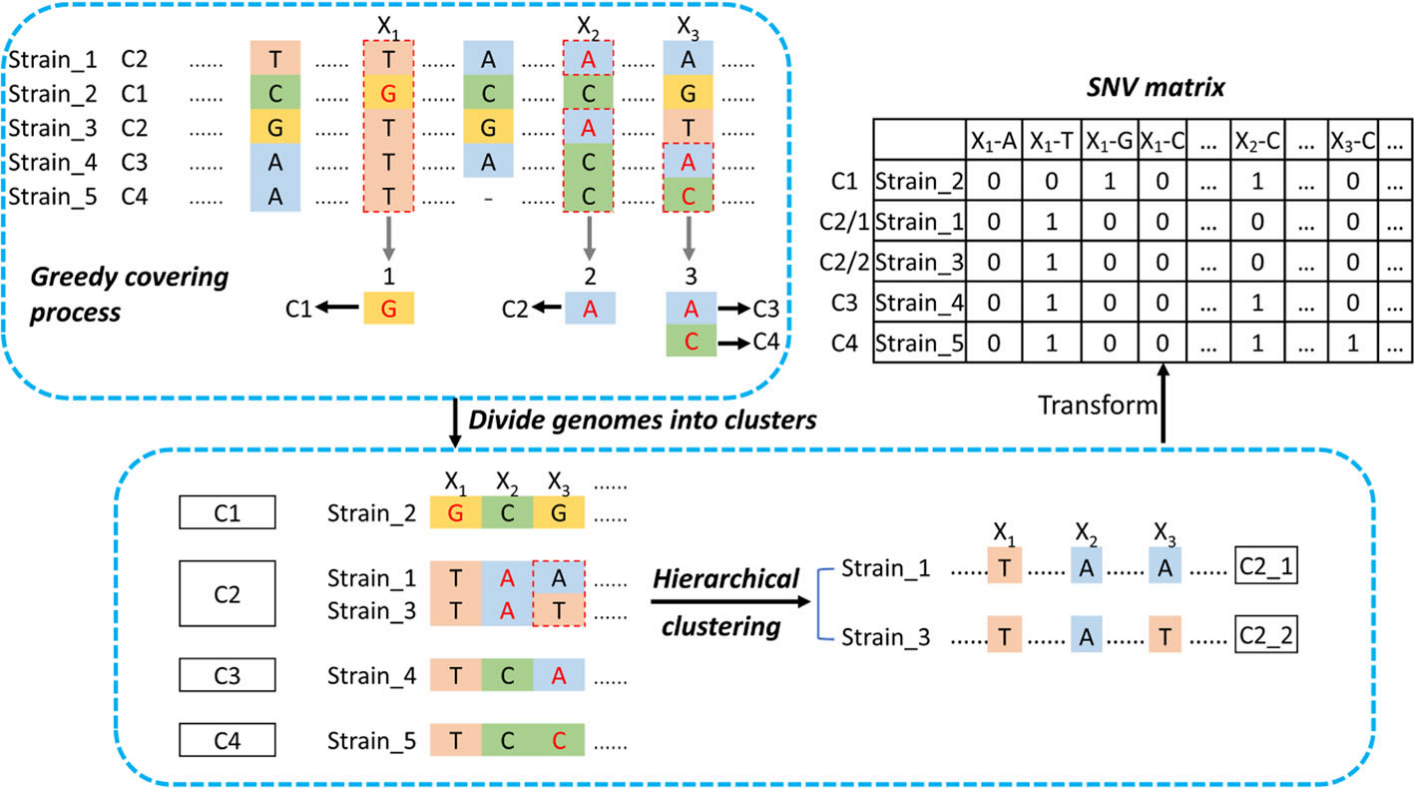
The sketch of the *k*-mer identification and SNV matrix construction stages. The SNVs in the matrix are represented by the site and base [18]. “C1-C4” represents the name of clusters

Then, we pick the column with minimum positive $\mathcal {H}$ from $\mathcal {M}$. Let this column be *s*_*i*_. Let the nucleotide at *s*_*i*_ with the minimum frequency be $s^{min}_{i}$. All the genomes containing $s^{min}_{i}$ at site *s*_*i*_ will be extracted and saved in one cluster. The entropy for the remaining genomes will be updated after the extraction. And this greedy choice will be applied to the remaining genomes until all the genomes are in one cluster. It should be noted that low-quality columns with too many dashes will not be considered and can be filtered in pre-processing. Depending on the reference genome similarity and alignment quality, users can choose a threshold for the allowed percentage of dashes in one column. For SARS-CoV-2 and H1N1(HA), our default cutoff is 0. For HIV, our default cutoff is 10%. The pseudocode of the entropy-based greedy covering algorithm is presented in Algorithm 1. A working example is shown in the top-left panel of Fig. 9.

**Fig. 10:**
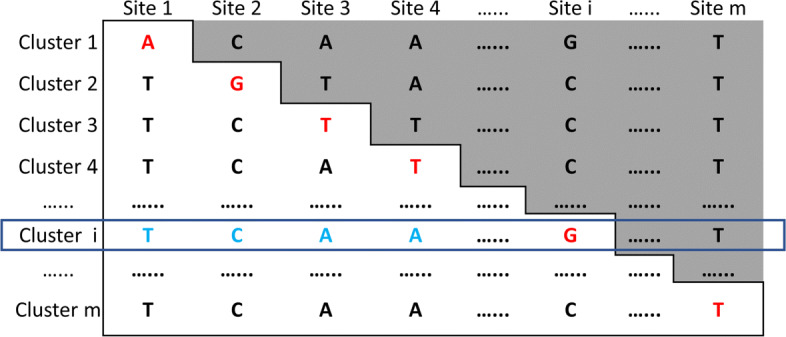
The SNV sites and corresponding clusters. Site *i* is the SNV site chosen at the *i*th step. The base in red denotes the base with the minimum *p*^*b*^ (i.e. $s^{min}_{i}$). Genomes in the same cluster share the red base at the chosen SNV site. Each cluster has unique SNV base combination, which is shown in the white part of each row. The SNV base combination for cluster *c*_*i*_ is shown in a box

After we apply this greedy covering algorithm, the reference genomes are divided into multiple clusters, where each cluster is defined by one SNV event. Figure 10 sketches the SNV sites for different clusters based on the order of SNV site selection in the greedy covering algorithm. Let the number of chosen SNV sites (i.e., the final number of clusters) be *m*. Let *s*_*i*_ be the SNV site chosen at the *i*th step in the greedy covering algorithm. Again, $s^{min}_{i}$ is the base with minimum frequency at site *s*_*i*_ in the remaining genomes. Let the corresponding cluster be *c*_*i*_, which contains the genomes containing $s^{min}_{i}$ at the *i*th step. We use $s^{min}_{i}$ at SNV sites *s*_1_, *s*_2_, …, *s*_*i*_ to represent cluster *c*_*i*_. We have the following theorem and proof.

#### **Theorem 1**

Let the SNV base combination for the *ith* cluster *c*_*i*_ be ${s^{min}_{1}, s^{min}_{2}, \ldots, s^{min}_{i}}$. This nucleotide base combination uniquely represent the genomes in cluster *c*_*i*_.

#### *Proof*

In order to prove that the nucleotide base combination ${s^{min}_{1}, s^{min}_{2}, \ldots, s^{min}_{i}}$ uniquely represent the genomes in cluster *c*_*i*_, we need to show that at least one base of these SNV events in *c*_*i*_ is different from any other cluster *c*_*j*_, where *j*≠*i*. Figure 10 can be used to illustrate this proof.

Without losing generality, we consider two cases. In case 1, we consider a cluster *c*_*j*_ with *j*<*i*. The bases at *j* in genomes of *c*_*j*_ must be identical (condition for clustering). In addition, that base must be different from the base at site *s*_*j*_ in genomes of cluster *c*_*i*_. Otherwise, the genomes of *c*_*i*_ will be clustered into *c*_*j*_ at step *j*. In the second case, we consider a cluster *c*_*j*_ with *j*>*i*. For any genomes in *c*_*j*_, their base at SNV site *s*_*i*_ must be different from the base at site $s^{min}_{i}$ of the genomes in *c*_*i*_. Otherwise, that genome will be clustered into *c*_*i*_. Thus, we proved that at least one base at the SNV event combination in *c*_*i*_ is different from any other cluster *c*_*j*_.

Thus, as shown by Fig. 10, we identified unique SNV combinations for each cluster. As we found the columns involving indels may have alignment errors or assembly errors (an example can be found in Supplementary File 1, Supplementary Figure S6), we only use columns with no gap or a small number of gaps in greedy covering. As a result, some clusters can contain multiple genomes. In this case, the genomes inside each cluster can be aligned again (to reduce the alignment errors) and be clustered in a hierarchical fashion. An example can be found in the bottom panel of Fig. 9.

#### Maintaining “balanced” SNV site combinations

In the ideal case of no sequencing errors and each base of a viral genome being covered by at least one read, the SNV site sets that uniquely represent genomes in each cluster *c*_*i*_ are sufficient to determine the cluster or the strain precisely. But in reality, both heterogeneous coverage and sequencing errors exist. For clusters represented by a small number of SNV sites (e.g., *c*_1_ contains just one SNV site), sequencing errors can incur false positives. To address this issue, we will balance the number of the SNV sites for each cluster so that each cluster has the same number of SNV sites. To do so, we will use all *m* SNV sites for strain identification. If the original SNV sites can distinguish the genomes in different clusters, adding SNV sites will not change this property. Thus, each cluster still possesses unique SNV site combination. As shown in Fig. 10, the SNV bases in both the white and gray part will be used for strain identification.

### Step 2: iterative strain search algorithm

#### *k*-mer extraction

We will extract *k*-mer from these SNV sites, with the center base of each *k*-mer coming from this site (see Supplementary File 1, Supplementary Section 2.1). Supplementary File 1, Supplementary Figure S7 shows an example of *k*-mer extraction. In order to avoid using *k*-mer that repeat at different sites in the MSA, we determine the *k*-mer size by examining the repeat times of *k*-mer of different *k* in the MSA. We find that with the increase of *k*, the repeat numbers of the *k*-mer at different sites reduce quickly (Supplementary File 1, Supplementary Figure S8). By default, we use *25*-mer.

**Fig. 11:**
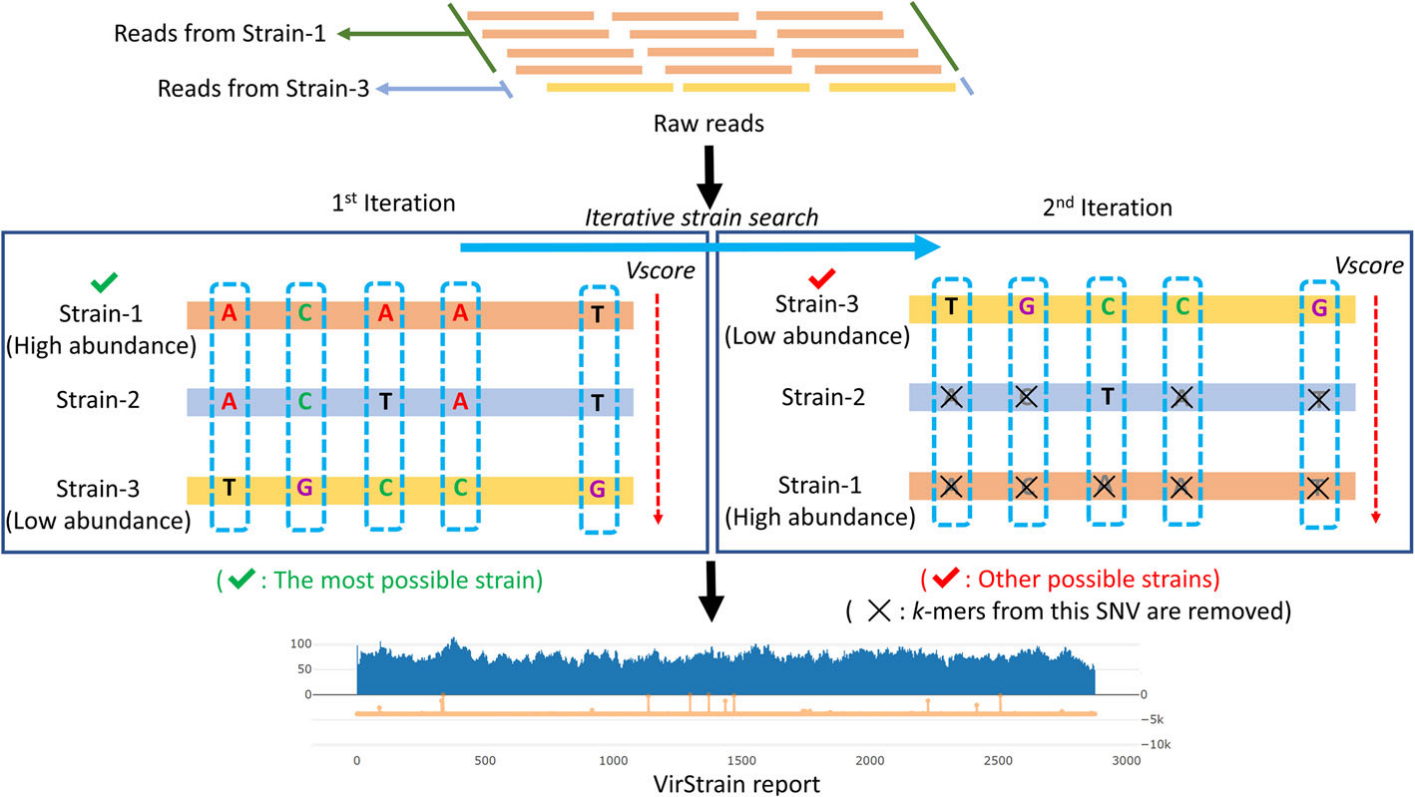
The overall workflow of the iterative strain identification. The red arrow represents that all strains are listed in descending order of *Vscore*

To detect all possible strains in a sample, we take an iterative strategy similar to QuantTB [18]. The overall workflow of the strain search algorithm is displayed in Fig. 11. For an input set of reads, the *k*-mer match frequencies are computed using a *k*-mer counting tool and are mapped to an SNV matrix, which will allow us to quickly compute the sum of the coverage for all the SNV sites and rank the strains. The major operations are described below.

#### Construction of the SNV matrix

The SNV sites chosen by the greedy covering algorithm will be used to construct an SNV matrix *S* of size 4*mn*, where *m* is the number of chosen variation sites and *n* is the number of reference genomes. An example is given in Fig. 9. For a strain *i* and a chosen SNV site *x*, there are four cells corresponding to bases A, C, G, and T in *S*. Denote an SNV event as *x*-*b*, indicating that base *b* is observed at site *x*. A cell *S*_*i*,*x*−*b*_ is 1 if the strain *i* has base *b* at site *x*. Otherwise, it is 0. Each cell in the matrix *S* has associated *k*-mer match frequency.

#### Rank the reference genomes using *k*-mer match frequency

We apply Jellyfish (V2.3.0) [58], a fast multi-threaded *k*-mer counter, to count *k*-mer in the sequencing data. Let *F*_*x*−*b*_ be the *k*-mer match numbers of base *b* at site *x*. Thus, *S*_*i*,*x*−*b*_=*S*_*i*,*x*−*b*_∗*F*_*x*−*b*_. Then, we will compute the frequency of base *b* at site *x* by normalize the *F*_*x*−*b*_. Therefore, $S_{i,x-b} = \frac {S_{i,x-b}}{\sum _{b \in \Sigma } S_{i,x-b}}$. To reduce the effect of sequencing error, we filter *S*_*i*,*x*−*b*_ if its value is smaller than a given threshold.

Once *S* is updated based on the actual *k*-mer match frequency from the reads, we will compute the score of strain *i* using $vscore_{i} = \frac {\sum _{x=1\ldots m} S_{i, x-b}}{\sum _{x=1\ldots m} I(S_{i,x-b}>0)}$, where *I* is an identity function. *vscore* favors strains with the most number of *k*-mer hits. Although it looks reasonable to consider other factors such as uniformity of *k*-mer match frequency, our empirical studies show that considering the total number of *k*-mer hits renders the best accuracy. One possible reason is the heterogeneous coverage of real sequencing data along RNA viral genomes. Read coverage profiles of 11 real sequencing datasets in our experiment can be found in Supplementary File 1, Supplementary Figure S9. We will compute *vscore*_*i*_ for all the strains and rank them in decreasing order.

#### Iterative strain search

VirStrain takes an iterative approach to search for multiple strains. VirStrain will output the top 1 strain in the ranked list and then update *S* by replacing the frequency of all the SNV sites in identified strain with 0. Any strains that share the same SNV bases with the identified strains cannot reuse the frequency. Otherwise, strains that share high similarity with the identified ones can easily get higher *Vscore* than low-abundance strains that are not similar to the best match. An example is given in Fig. 11. At each iteration, the sequencing depth is calculated by taking the average frequency of its SNVs for each identified strain in the sample. VirStrain continues to calculate the score and identifies the best matched strain in each iteration until the frequency values of all variations become 0. At each iteration, the sequencing coverage of the identified strain is calculated by taking the average of the *k*-mer match frequencies. In the end, this iterative process will return a list of strains with their *k*-mer coverage profiles on the SNV sites.

We sacrifice the resolution of finding highly similar strains in the same sample by avoiding introducing false-positive hits via the iterative search strategy. If there are indeed highly similar strains such as those in quasispecies, the most abundant one will be output as a representative. We conducted experiments to examine how many different SNV sites are needed for VirStrain to recognize multiple strains (see Supplementary File 1, Supplementary Section 1.11).

## Supplementary Information

**Additional file 1** Supplementary information.

**Additional file 2** Review history.

## Data Availability

All command lines running tools in this work can be found in the GitHub repository (https://github.com/liaoherui/ToolsCommandLines). The source code of VirStrain is freely available at https://github.com/liaoherui/VirStrain, under MIT license. The versions used in the manuscript are permanently available at 10.5281/zenodo.5700305 [59]. Datasets used in this paper are all publicly available. The simulated datasets of SARS-CoV-2, H1N1, and HIV are available at 10.5281/zenodo.5810419[60]. All SARS-CoV-2 single-strain sequencing datasets used in this manuscript can be downloaded from the NCBI SRA database (https://www.ncbi.nlm.nih.gov/sra) [61] with accession numbers: SRR10971381, SRR11593364, SRR11880659, SRR13644068, SRR12546786, ERR4387386, SRR12528370, SRR11513776, SRR11570921, SRR13634208, SRR11593362, SRR11593358, SRR13574086, SRR13574250, SRR13574082, SRR13644095, SRR13718002, SRR13684392, SRR13644074, SRR13681138, SRR13684393, SRR13499395, SRR13499389, SRR13499360, SRR11968882, SRR12316191, SRR12588591, SRR12598969, SRR12598968, SRR12352751, SRR12352750, and SRR11587603. The SARS-CoV-2 mix-strain sequencing datasets are downloaded from [48] (SRA accession number: SRR14142136 and SRR14142137). The HIV 5-strain mock data is downloaded from [49] (SRA accession number: SRR961514). The HBV mix-strain sequencing datasets are downloaded from [50] (SRA accession number: ERR3253398 and ERR3253399). All HCMV mock datasets are downloaded from [51] (NCBI BioProject: PRJEB32127). All HCMV mix-strain datasets are downloaded from [52] (NCBI BioProject: PRJNA605798). Andrew Cosgrove was the primary editor of this article and managed its editorial process and peer review in collaboration with the rest of the editorial team. The review history is available as Additional file 2.

## References

[1] Kiso M (2004). Resistant influenza A viruses in children treated with oseltamivir: descriptive study. Lancet.

[2] Perrin L, Telenti A (1998). HIV treatment failure: testing for HIV resistance in clinical practice. Science.

[3] Hadfield J (2018). Nextstrain: real-time tracking of pathogen evolution. Bioinformatics.

[4] Ladner J (2019). Precision epidemiology for infectious disease control. Nat Med.

[5] Gudbjartsson D (2020). Spread of SARS-CoV-2 in the Icelandic Population. N Engl J Med.

[6] Yan Y (2020). Strain-level epidemiology of microbial communities and the human microbiome. Genome Med.

[7] Kuhn J (2013). Virus nomenclature below the species level: a standardized nomenclature for natural variants of viruses assigned to the family Filoviridae. Arch Virol.

[8] Islam M, et al.Genome-wide analysis of SARS-CoV-2 virus strains circulating worldwide implicates heterogeneity. Sci Rep. 2020; 10(14004). 10.1038/s41598-020-70812-6 PMC7438523 32814791

[9] Ahn T, Chai J, Pan C (2015). Sigma: strain-level inference of genomes from metagenomic analysis for biosurveillance. Bioinformatics.

[10] Harel N (2019). Direct sequencing of RNA with MinION Nanopore: detecting mutations based on associations. Nucleic Acids Res.

[11] Hong C (2014). PathoScope 2.0: a complete computational framework for strain identification in environmental or clinical sequencing samples. Microbiome.

[12] Wood D, Salzberg S (2014). Kraken: ultrafast metagenomic sequence classification using exact alignments. Genome Biol.

[13] Chen S, et al.A computational toolset for rapid identification of SARS-CoV-2, other viruses and microorganisms from sequencing data. Brief Bioinforma. 2020. 10.1093/bib/bbaa231 PMC7543257 33003197

[14] Roosaare M (2017). StrainSeeker: fast identification of bacterial strains from raw sequencing reads using user-provided guide trees. PeerJ.

[15] Neher R, Bedford T (2015). nextflu: real-time tracking of seasonal influenza virus evolution in humans. Bioinformatics.

[16] Chen J, Huang J, Sun Y (2019). TAR-VIR: a pipeline for TARgeted VIRal strain reconstruction from metagenomic data. BMC Bioinforma.

[17] Truong D (2017). Microbial strain-level population structure and genetic diversity from metagenomes. Genome Res.

[18] Anyansi C (2020). QuantTB - a method to classify mixed Mycobacterium tuberculosis infections within whole genome sequencing data. BMC Genomics.

[19] Rose R, Constantinides B, Tapinos A, Robertson D, Prosperi M (2016). Challenges in the analysis of viral metagenomes. Virus Evol.

[20] Posada-Cespedes S, Seifert D, Beerenwinkel N (2017). Recent advances in inferring viral diversity from high-throughput sequencing data. Virus Res.

[21] Chen J, Zhao Y, Sun Y (2018). De novo haplotype reconstruction in viral quasispecies using paired-end read guided path finding. Bioinformatics.

[22] Eliseev A, Gibson K (2020). Evaluation of haplotype callers for next-generation sequencing of viruses. Infect Genet Evol.

[23] Knyazev S, Hughes L, Skums P, Zelikovsky A (2021). Epidemiological data analysis of viral quasispecies in the next-generation sequencing era. Brief Bioinform.

[24] Knyazev S (2021). Accurate assembly of minority viral haplotypes from next-generation sequencing through efficient noise reduction. Nucleic Acids Res.

[25] Skittrall J (2019). A scale-free analysis of the HIV-1 genome demonstrates multiple conserved regions of structural and functional importance. PLoS Comput Biol.

[26] Alves B (2019). Estimating HIV-1 genetic diversity in Brazil through next-generation sequencing. Front Microbiol.

[27] Bao Y, Chetvernin V, Tatusova T (2014). Improvements to pairwise sequence comparison (PASC): a genome-based web tool for virus classification. Arch Virol.

[28] Muhire B, Varsani A, Martin D (2014). SDT: a virus classification tool based on pairwise sequence alignment and identity calculation. PLoS ONE.

[29] Huang W (2012). ART: a next-generation sequencing read simulator. Bioinformatics.

[30] Shu Y, McCauley J (2017). GISAID: Global initiative on sharing all influenza data - from vision to reality. Euro Surveill.

[31] Wood D (2019). Improved metagenomic analysis with Kraken 2. Genome Biol.

[32] Breitwieser F (2018). KrakenUniq: confident and fast metagenomics classification using unique k-mer counts. Genome Biol.

[33] Kim D (2016). Centrifuge: rapid and sensitive classification of metagenomic sequences. Genome Res.

[34] Dilthey A (2019). Strain-level metagenomic assignment and compositional estimation for long reads with MetaMaps. Nat Commun.

[35] Zhang Z (2000). A greedy algorithm for aligning DNA sequences. J Comput Biol.

[36] Zagordi O, Bhattacharya A (2011). ShoRAH: estimating the genetic diversity of a mixed sample from next-generation sequencing data. BMC Bioinforma.

[37] Prabhakaran S, Rey M (2014). HIV haplotype inference using a propagating Dirichlet process mixture model. IEEE/ACM Trans Comput Biol Bioinform.

[38] Ahn S, Vikalo H (2018). aBayesQR: a Bayesian method for reconstruction of viral populations characterized by low diversity. J Comput Biol.

[39] Ahn S, Ke Z, Vikalo H (2018). Viral quasispecies reconstruction via tensor factorization with successive read removal. Bioinformatics.

[40] Abdou Chekaraou M (2010). A novel hepatitis B virus (HBV) subgenotype D (D8) strain, resulting from recombination between genotypes D and E, is circulating in Niger along with HBV/E strains. J Gen Virol.

[41] Hu Y (2017). Identification of two new HIV-1 circulating recombinant forms (CRF87_cpx and CRF88_BC) from reported unique recombinant forms in Asia. AIDS Res Hum Retroviruses.

[42] Pang J, et al.Haplotype assignment of longitudinal viral deep-sequencing data using co-variation of variant frequencies. bioRxiv. 2020;:444877. 10.1093/ve/veac093 PMC9719071 36478783

[43] Yue J, Liti G (2019). simuG: a general-purpose genome simulator. Bioinformatics.

[44] van Dorp L (2020). Emergence of genomic diversity and recurrent mutations in SARS-CoV-2. Infect Genet Evol.

[45] Li D (2015). MEGAHIT: an ultra-fast single-node solution for large and complex metagenomics assembly via succinct de Bruijn graph. Bioinformatics.

[46] Langmead B, Salzberg S (2012). Fast gapped-read alignment with bowtie 2. Nat Methods.

[47] Wu F (2020). A new coronavirus associated with human respiratory disease in China. Nature.

[48] Samoilov A (2021). Case report: change of dominant strain during dual SARS-CoV-2 infection. BMC Infect Dis.

[49] Giallonardo F (2014). Full-length haplotype reconstruction to infer the structure of heterogeneous virus populations. Nucleic acids Res.

[50] McNaughton A (2019). Illumina and Nanopore methods for whole genome sequencing of hepatitis B virus (HBV). Sci Rep.

[51] Deng Z, Dhingra A, et al.Evaluating assembly and variant calling software for strain-resolved analysis of large DNA viruses. Brief Bioinforma. 2021; 22(3). 10.1093/bib/bbaa123 PMC8138829 34020538

[52] Pang J (2020). Mixed cytomegalovirus genotypes in HIV-positive mothers show compartmentalization and distinct patterns of transmission to infants. Elife.

[53] Richardson B (2016). Vertical cytomegalovirus transmission from HIV-infected women randomized to formula-feed or breastfeed their infants. J Infect Dis.

[54] Katoh K (2002). MAFFT: a novel method for rapid multiple sequence alignment based on fast Fourier transform. Nucleic Acids Res.

[55] Price M, Dehal P, Arkin A (2010). FastTree 2–approximately maximum-likelihood trees for large alignments. PLoS ONE.

[56] Letunic I, Bork P (2019). Interactive Tree Of Life (iTOL) v4: recent updates and new developments. Nucleic Acids Res.

[57] Suárez NM (2019). Multiple-strain infections of human cytomegalovirus with high genomic diversity are common in breast milk from human immunodeficiency virus-infected women in Zambia. J Infect Dis.

[58] Marcais G, Kingsford C (2011). A fast, lock-free approach for efficient parallel counting of occurrences of k-mers. Bioinformatics.

[59] Herui L, Yanni S, Dehan C. liaoherui/VirStrain: First Release of VirStrain. 10.5281/zenodo.5700305.

[60] Herui L, Dehan C, Yanni S. The Simulated Datasets Used in VirStrain’s Paper. 10.5281/zenodo.5810419.

[61] Kodama Y, Shumway M, Leinonen R (2012). The Sequence Read Archive: explosive growth of sequencing data. Nucleic Acids Res.

