# Supplementary material for: VirStrain: a strain identification tool for RNA viruses
Source: Genome Biol. 2022 Jan 31;23:38. doi: 10.1186/s13059-022-02609-x (PMC8801933; doi:10.1186/s13059-022-02609-x)
Supplement: Supplementary file 1 — Additional file 1 Supplementary information. [file 13059_2022_2609_MOESM1_ESM.pdf]

Supplementary information for “VirStrain: a strain identification tool for RNA viruses”

Herui Liao, Dehan Cai, and Yanni Sun

Electrical Engineering Department, City University of Hong Kong, Kowloon, Hong Kong SAR

Jan 2022

1 Supplementary Experiments

1.1 The impact of the database size on the performance of VirStrain

To exam whether the number of reference genomes affects the performance of VirStrain, we build two databases with different numbers of reference genomes and repeat the experiment mentioned in Section 2.3 of the main article. These two databases contain 6880 and 3447 SARS-CoV-2 strains, respectively. The strains used to build the database with 6880 strains are divided into two parts. One part is the same strains from NCBI as the main article, and the other part is newly added strains from GISAID [1]. The newly added SARS-CoV-2 strains are downloaded according to their GISAID accession IDs in nextstrain’s [2] SARS-CoV-2 global subsampling page. The database with 3447 strains is constructed using strains from GISAID. The result is shown in Supplementary Table S1. VirStrain has a slight improvement at lower coverage when there are fewer SARS-CoV-2 strains in the database. The number of multiple hits may increase when the number of reference genome increases and the sequencing depth decreases.

		75bp	100bp	150bp	250bp
Database with 6880 SARS-CoV-2 strains	5x	(1111, 7)	(928, 6)	(742, 4)	(805, 4)
	10x	(159, 2)	(117, 2)	(62, 2)	(67, 2)
	20x	(0, 1)	(0, 1)	(0, 1)	(0, 1)
	50x	(0, 1)	(0, 1)	(0, 1)	(0, 1)
	100x	(0, 1)	(0, 1)	(0, 1)	(0, 1)
Database with 4082 SARS-CoV-2 strains (Same as Table 1)	5x	(903, 7)	(782, 6)	(602, 5)	(679, 5)
	10x	(141, 2)	(109, 2)	(51, 2)	(65, 2)
	20x	(0, 1)	(0, 1)	(0, 1)	(0, 1)
	50x	(0, 1)	(0, 1)	(0, 1)	(0, 1)
	100x	(0, 1)	(0, 1)	(0, 1)	(0, 1)
Database with 3347 SARS-CoV-2 strains	5x	(730, 4)	(649, 3)	(579, 3)	(658, 4)
	10x	(86, 2)	(99, 2)	(71, 2)	(49, 2)
	20x	(0, 1)	(0, 1)	(0, 1)	(0, 1)
	50x	(0, 1)	(0, 1)	(0, 1)	(0, 1)
	100x	(0, 1)	(0, 1)	(0, 1)	(0, 1)

Supplementary Table S1. The number of tie cases and the median number of best matches for the tie cases in VirStrain. There are 3 databases containing different numbers of reference genomes. Each cell contains a tuple with the first number being the number of tie cases and the second number being the median number of strains in the top-ranking group.

1.2 Benchmark experiment on all strains

In order to test the performance of VirStrain on all strains, we carried out a benchmark experiment with VirStrain, KrakenUniq, and Centrifuge. As Sigma and Pathoscope2 are more computationally expensive than other tools, they are not compared in this experiment. Kraken2 is also not compared due to its low accuracy on the single-strain dataset.

In this experiment, one of the SARS-CoV-2 strains in the reference database was randomly selected as the test strain. We simulated reads from it and used the reads as input to the three tools. This process is repeated for all other strains. The accuracy is computed for all the strains and shown in Supplementary Figure S3. VirStrain is able to identify all strains in the database while other tools have lower accuracy.

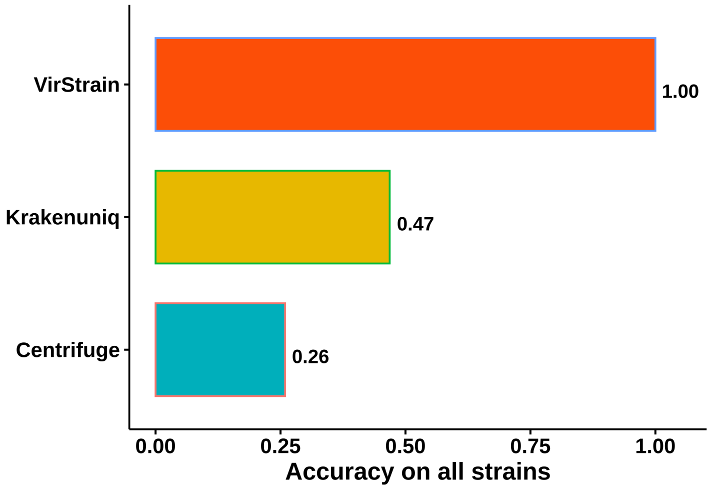

Supplementary Figure S1. The accuracy comparison of 3 tools on all strains in the database. These three tools have fast speed and also reasonable accuracy in the small scale experiment on 200 reference genomes.

1.3 Benchmark experiments on low coverage data

In order to test the performance of other tools at low sequencing depth, we evaluate other methods (Sigma, Pathoscope2, Centrifuge, Krakenuniq) on the same datasets used in Table 1 of the main article. Due to the long running time of Pathoscope2 and Sigma, we only utilized 200 strains as their reference database. The result is summarized in Supplementary Table S2, which shows the major problem of most tested tools is the low accuracy. When the sequencing depth decreases, the accuracy of Centrifuge, Krakenuniq, and Pathoscope2 decreases as well. Besides, the two computationally efficient tools, Centrifuge and Krakenuniq, also have many multiple hits when the sequencing depth is low. Though Sigma has a good performance in this evaluation, it took more than two weeks to analyze all datasets given 8 threads on an HPCC CentOS 6.8 node with 2.4Ghz 14-core Intel Xeon E5-2680v4 CPUs and 128 GB memory. On top of that, it has low accuracy in the mix-strain identification experiments (Figure 3A).

1.4 In-depth analysis of the 6 haplotype reconstruction tools

All haplotypes investigated in this experiment refer to the haplotypes falsely constructed by 6 haplotype reconstruction tools in Section 2.5 of the main article. For each haplotype, we used MegaBLAST [3] to identify the closest reference strain (defined as s) in the database. Then, we identified the reference strains that have higher similarity to the ground truth than s. The number of these reference strains is shown in Supplementary Table S3. For "missed strains" in Table S3, there are four cases: 1) the dataset contains two strains but the tool only outputs one; 2) a tool outputs two haplotypes but their s is the same strain; 3) the haplotype cannot be aligned to any reference strain; 4) the tool outputs nothing. Because of the high divergence of the HIV strains, all tools perform better on HIV. ShoRAH and PredicHaplo tend to miss more strains than other tools. In particular, PredictHaplo failed to output any haplotype for a large number of samples. Many outputs of ShoRAH cannot be aligned to any reference genome. aBayesQR and TenSQR can output two haplotypes in more datasets than other tools. TenSQR outputs haplotypes that are closer to

		75bp	100bp	150bp	250bp
VirStrain	5x	(1.000, 903, 7)	(1.000, 782, 6)	(1.000, 602, 5)	(1.000, 679 ,5)
	10x	(1.000, 141, 2)	(1.000, 109, 2)	(1.000, 51, 2)	(1.000, 65, 2)
	20x	(1.000, 0, 1)	(1.000, 0, 1)	(1.000, 0, 1)	(1.000, 0, 1)
	50x	(1.000, 0, 1)	(1.000, 0, 1)	(1.000, 0, 1)	(1.000, 0, 1)
	100x	(1.000, 0, 1)	(1.000, 0, 1)	(1.000, 0, 1)	(1.000, 0, 1)
Centrifuge	5x	(0.620, 131, 2)	(0.608, 120, 2)	(0.560, 115, 2)	(0.333, 77 ,2)
	10x	(0.656, 151, 2)	(0.653, 151, 2)	(0.641, 142, 2)	(0.504, 88, 2)
	20x	(0.661, 155, 2)	(0.661, 150, 2)	(0.664, 155 ,2)	(0.618, 128, 2)
	50x	(0.648, 144, 2)	(0.658, 150, 2)	(0.663, 155, 2)	(0.657, 141, 2)
	100x	(0.646, 133, 2)	(0.658, 137, 2)	(0.664, 143, 2)	(0.662, 146, 2)
Krakenuniq	5x	(0.468, 61, 14)	(0.472, 92, 15)	(0.467, 94, 18)	(0.462, 126, 2)
	10x	(0.477, 22, 22)	(0.474, 17, 4)	(0.473, 17, 3)	(0.471, 28, 3)
	20x	(0.478, 11, 46)	(0.476, 11, 2)	(0.475, 12, 3)	(0.473, 12 ,6)
	50x	(0.480, 9, 6)	(0.480, 15, 6)	(0.477, 4, 3)	(0.476, 9, 2)
	100x	(0.482, 10, 2)	(0.481, 10, 2)	(0.481, 7, 2)	(0.479, 6, 2)
Pathoscope2	5x	(0.300, 0 ,1)	(0.315, 0 ,1)	(0.365, 0, 1)	(0.315, 0, 1)
	10x	(0.465, 0, 1)	(0.480, 0, 1)	(0.475, 0 ,1)	(0.400, 0 ,1)
	20x	(0.610, 0, 1)	(0.630, 0, 1)	(0.660, 0, 1)	(0.635, 0, 1)
	50x	(0.795, 0, 1)	(0.805, 0, 1)	(0.790, 0, 1)	(0.765, 0 ,1)
	100x	(0.910, 0, 1)	(0.865, 0, 1)	(0.885, 0, 1)	(0.840, 0 , 1)
Sigma	5x	(0.995, 0, 1)	(1.000, 0, 1)	(1.000, 1, 2)	(0.990, 0, 1)
	10x	(1.000, 0, 1)	(1.000, 0, 1)	(1.000, 0, 1)	(1.000, 0, 1)
	20x	(1.000, 0, 1)	(1.000, 0, 1)	(1.000, 0, 1)	(1.000, 0, 1)
	50x	(1.000, 0, 1)	(1.000, 0, 1)	(1.000, 0, 1)	(1.000, 0, 1)
	100x	(1.000, 0, 1)	(1.000, 0, 1)	(1.000, 0, 1)	(1.000, 0, 1)

Supplementary Table S2. The accuracy, number of tie cases, and the median number of best matches in all tie cases of 5 tools. VirStrain, Centrifuge, and Krakenuniq are tested on the 2,280 $\times$ 20 simulated datasets. It should be noted there are only 200 $\times$ 20 simulated datasets for Sigma and Pathoscope2 due to their long running time. Nevertheless, Sigma took more than two weeks to analyze these 200 $\times$ 20 simulated datasets. Each cell contains three numbers: The first number being accuracy, the second number being the number of tie cases, and the third number being the median number of strains in the top-ranking group.

the ground truth than aBayesQR. Furthermore, we plotted the similarity distribution between these haplotypes and the ground truth in Supplementary Figure S4. Because the strain-level divergence is different for different viruses, we should only compare tools for each type of virus separately. Although CliqueSNV can miss many strains, the ones it constructed are either the ground truth or highly similar to the ground truth. HaROLD has a higher similarity to the ground truth for SARS-CoV-2 than other viruses. aBayesQR and TenSQR have relatively stable performance across the three viruses. ShoRAH has only a small number of strains for computing the similarities because it missed many strains.

Virus	Tools	# of missed strains	# of strains (with 1-10 strains more similar to the ground truth)	# of strains (with 11-100 strains more similar to the ground truth)	# of strains (with >100 strains more similar to the ground truth)
SCOV2	aBayesQR	**50**	11	32	21
	CliqueSNV	109	**0**	1	2
	HaROLD	90	1	**0**	3
	PredictHaplo	145	**0**	**0**	1
	ShoRAH	200	**0**	**0**	**0**
	TenSQR	55	11	8	11
H1N1	aBayesQR	**82**	10	17	13
	CliqueSNV	128	1	15	7
	HaROLD	134	14	5	10
	PredictHaplo	199	**0**	**0**	**0**
	ShoRAH	200	**0**	**0**	**0**
	TenSQR	86	10	17	19
HIV	aBayesQR	**0**	4	**0**	**0**
	CliqueSNV	7	**1**	**0**	**0**
	HaROLD	1	9	3	5
	PredictHaplo	178	**1**	**0**	**0**
	ShoRAH	110	3	31	54
	TenSQR	**0**	2	**0**	**0**

Supplementary Table S3. The statistics of falsely constructed haplotypes. Each value in the cell refers to the number of haplotypes in that category. For example, in the first row, "50" means there are 50 SARS-CoV-2 haplotypes missed by aBayesQR. "11" means there are 11 SARS-CoV-2 haplotypes that can be aligned to 1-10 strains in the reference database with higher similar to the ground truth. For each virus and category, the bold number represents the best metric value.

1.5 VirStrain identifies a recombinant strain from 3-strain simulated data

To test whether VirStrain can identify the recombinant strains from mixed strain infections, two mixed strain simulation datasets are generated. These two datasets contain three HBV and three HIV strains, respectively. Considering we don't know the exact parent strains of the recombinant strains and thus we constructed the dataset using strains from the parent strains' genotypes/subtypes. As a result, these selected strains contain both the recombinant strain and the strains in its parent genotypes/subtypes [4, 5]. The sequencing data are simulated according to a sequencing depth of 10X:10X:10X. As shown in Supplementary Table S4, VirStrain can identify both the recombinant strain and the strains in its parent genotypes/subtypes. The databases for HBV and HIV used here is the same as the one used in the main article.

1.6 A large-scale experiment of novel strain detection

To further test the robustness of VirStrain, we applied VirStrain to detect the closest relative in a larger simulated dataset. Sigma was not included in this experiment because of its long running time. We randomly selected 100 strains from all the reference genomes. Then we generated 500 mutant strains from these 100 strains with 5, 7, 9, 11, 13 random point mutations. With ART, we simulated 600 datasets (for 500 mutant strains and 100 reference strains) and used them as input to VirStrain. VirStrain can still identify correct closest relatives in all the 600 simulated datasets (Supplementary Figure S5).

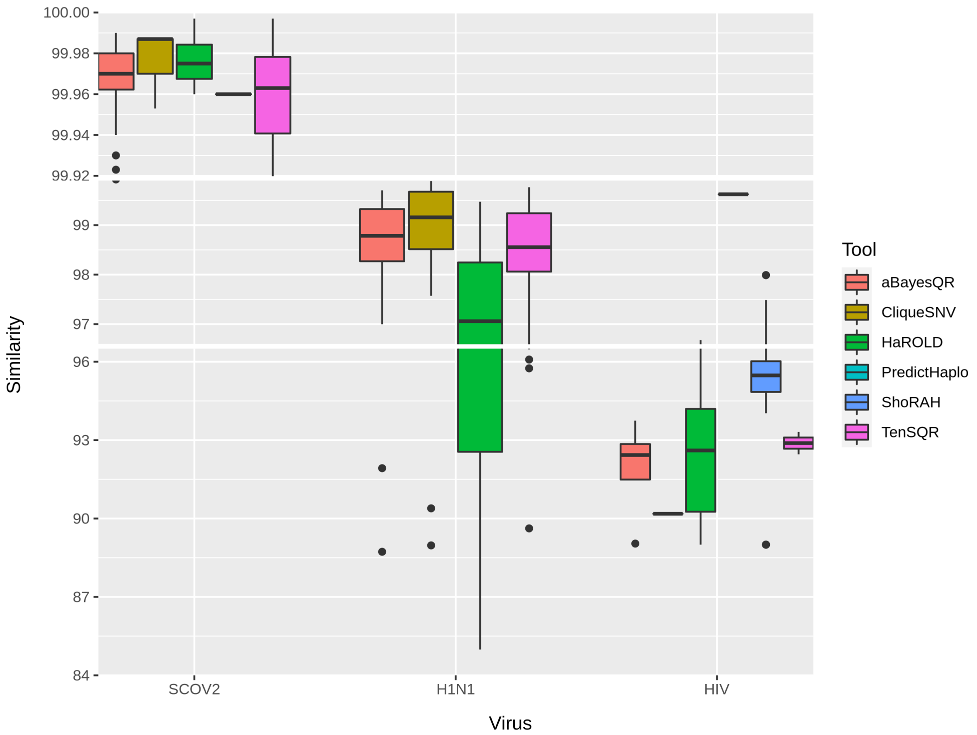

Supplementary Figure S2. The similarity between the falsely constructed haplotypes and the ground truth. The similarity is calculated by MegaBLAST.

Virus	Information of mix-strain simulated data			Strains detected	Rank	Predicted sequencing depth
	Reference	Depth	Description			
HBV	MT426115	10X	Genotype: E	Y	1	23.9
	MT426110	10X	Genotype: D	Y	3	11.25
	FN594771	10X	Genotype: DE (Recombinant strain)	Y	2	12.62
HIV	EF122545	10X	Subtype: B	Y	1	4.67
	KP234555	10X	Subtype: C	Y	2	4.38
	KF250402	10X	Subtype: BC (Recombinant strain)	Y	3	2.91

Supplementary Table S4. The performance of VirStrain on 2 mix-strain simulated datasets of HBV and HIV. "Rank" represents the ranking of each strain in the output of VirStrain.

1.7 The limitation of assembly tools

In this experiment, we apply 3 popular assembly tools to assemble viral genomes from 4 real SARS-CoV-2 sequencing data sets. As shown in Supplementary Table S5, these assembly tools are not able to generate the complete genomes from the input sequencing data, indicating that the whole virus genome may not always be available for downstream analysis. However, VirStrain can still be applied to these datasets to identify the most possible strain present in these samples. This experiment shows that VirStrain can be used for strain composition analysis when it is hard to obtain the complete genomes from the sequencing data.

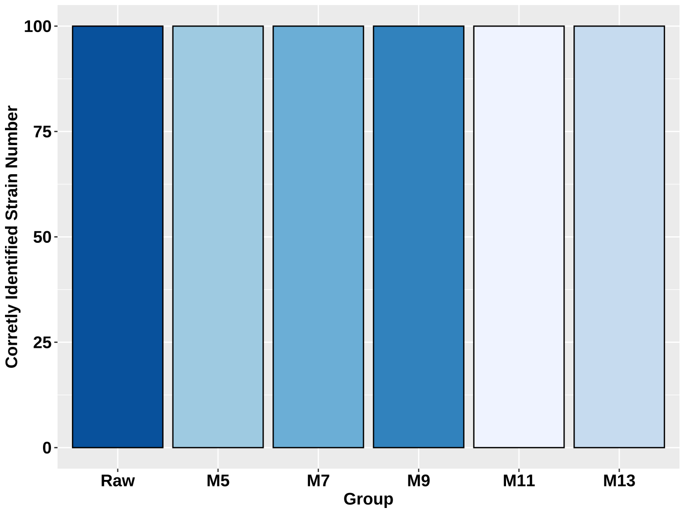

Supplementary Figure S3. The performance of VirStrain on detecting the closest relative on 600 simulated datasets. "Raw" means the original dataset and M5, M7, M9, M11, M13 represent datasets simulated from mutant strains. Each dataset contains one strain.

Datasets	Type	Megahit			Spades			IVA		
		N50	contigs	Largest contig (bp)	N50	contigs	Largest contig (bp)	N50	contigs	Largest contig (bp)
SAMN16068354	PE	672	362	2,501	646	250	2,616	748	50	1,375
SAMN16068353	PE	694	210	11,624	644	157	4,136	736	50	3,535
SAMN15678404	SE	1,127	69	9,686	1,006	86	13,630	-	-	-
SAMN15678405	SE	2,625	21	6,378	2,285	22	5,319	-	-	-

Supplementary Table S5. The performance of 3 assembly tools on 4 real sequencing data sets. "-" indicates that IVA is not tested on those datasets because it only accepts sequencing data of PE. The cell in grey means these datasets are metagenomic sequencing data.

1.8 Test four haplotype reconstruction tools using 2 HBV sequencing data sets with known composition

In this experiment, we applied 4 haplotype reconstruction tools to reconstruct strain sequences from 2 HBV mix-strain samples with known composition (MK720631.1 and MK720628.1). By using MegaBLAST [3], the reconstructed strain sequences are aligned to all strains in the reference database to find the most similar strain. As shown in Supplementary Table S6, the strains reconstructed by these tools are all most similar to the ground truth but are not 100% identical to the ground truth even though the similarity between the two strains is relatively low (89.97%).

Tools	ERR3253398		ERR3253399	
	MK720631.1	MK720628.1	MK720631.1	MK720628.1
HaROLD	(97.447, 73%)	(99.129, 27%)	**(98.042, 60%)**	(99.160, 40%)
aBayesQR	**(97.734, 45%)**	**(99.751, 22%)**	(97.300, 45%)	(97.357, 19%)
CliqueSNV	(96.706, 27%)	(98.383, 25%)	(96.551, 27%)	**(99.813, 38%)**
TenSQR	(97.733, 69%)	(99.533, 23%)	(97.919, 58%)	(99.782, 35%)

Supplementary Table S6. The performance of 4 haplotype reconstruction tools on the 2 HBV real samples (ERR3253398 and ERR3253399). Each cell contains a tuple with the first number being the similarity between the reconstructed strains and the ground truth and the second number being the relative abundance of the reconstructed strains. The reconstructed strains with the highest similarity to the ground truth are highlighted in bold.

1.9 VirStrain detects possible minor strains from Washington State datasets

For RNA viruses with fast replication rates, it is not rare to see multiple strains infecting the same host. For COVID-19, is it possible to see more than one strain (or haplotype) in the same host? Reconstructing the complete haplotype from short reads is an active research area itself and is beyond the scope of this work. But we can examine whether a different local haplotype segment exist in a sample. If we find one such local haplotype segment, we can add it to our reference sequence database and search for it in other samples using VirStrain. Being able to detect the same local haplotype in multiple samples provides strong evidence behind the presence of a new haplotype.

Towards this goal, we first assembled reads from sequenced SARS-CoV-2 samples at NCBI SRA using Megahit [6] and then mapped reads against the assembled genome. We found some relatively high-frequency mutations in a Washington State sample (SAMN15678406) from USA (Supplementary Figure S5). Based on the results of the reads mapping and a statistical model that considers the base quality, sequencing errors, coverage, and the number of reads covering the same SNV events, we obtained a possible local region of a minor strain, which we named as k59_23. We used nextstrain [2] to annotate k59_23 and found that there is a mutation G25358C that will cause the amino acid mutation D1259H of the surface protein. The statistical model used to find the possible local haplotype is described below.

There are four observed single nucleotide variants (SNVs) with frequencies much higher than sequencing errors at different loci of reference genome based on the read mapping results: 25345-A, 25355-C, 25357-C, 25358-C. We regard other types of SNVs at these four loci as sequencing errors because their frequencies are very low. We are not aiming to reconstruct the complete (genome-scale) haplotype, which is an active research field itself and is beyond the scope of this work. Instead, we will construct a local haplotype region containing SNV sites that are close enough to be covered by the same reads.

In the reference genome, the bases at the four loci are 25345-C, 25355-G, 25357-A, 25358-G. Thus, of the $2^4 - 1 = 15$ combinations of bases (excluding the reference one), our goal is to determine the most possible combination, which can define a local haplotype. For the sake of convenience, we use $k = 1, 2, 3, 4$ to denote these four loci. And we define the following notations: **a)** $q_k^{(j)}$ is the Phred quality score of the base in read j when this base mismatches the base of reference genome at locus k in the alignment between the read j and the reference genome. If the base in read j matches the base of reference genome at locus k in the alignment, or read j does not cover locus k in the alignment, the value of $q_k^{(j)}$ will be set to 0. **b)** $c_k^{(j)} \in \{0, 1\}$ is an indication function. $c_k^{(j)} = 1$ if the base in read j is not a sequencing error at locus k in the alignment. $c_k^{(j)} = 0$ if the base in read j is a sequencing error at locus k in the alignment, or read j does not cover locus k in the alignmen. **c)** m_k is the number of reads whose bases match the SNV at locus k in the alignments between the reads and the reference genome. **d)** d_k is the number of aligned reads that are aligned to locus k. **e)** $p_k^{(j)}$ is the probability that read j supports the SNV at locus k. And We use the equations(1) to calculate $p_k^{(j)}$.

$$p_k^{(j)} = \begin{cases} 1 - 10^{-\frac{q_k^{(j)}}{10}} & \text{if } q_k^{(j)} \neq 0 \; and \; c_k^{(j)} = 1 \\ 0 & \text{if } q_k^{(j)} = 0 \; and \; c_k^{(j)} = 1 \\ 10^{-\frac{q_k^{(j)}}{10}} \times \frac{m_k}{d_k} & \text{if } q_k^{(j)} \neq 0 \; and \; c_k^{(j)} = 0 \\ \frac{m_k}{d_k} & \text{if } q_k^{(j)} = 0 \; and \; c_k^{(j)} = 0 \end{cases} \tag{1}$$

There are four cases in Equation (1), also as shown in Figure S1. **In case 1**, the base in read j aligned to locus k of the reference genome matches the SNV at locus k. In this case we can use the Phred quality score to calculate the SNV probability directly. **In case 2**, the base in read j aligned to locus k of the reference genome matches the base in the reference genome and thus this read does not support the SNV event. So it is reasonable to assume that the probability of read j containing SNV at locus k is 0. **In case 3**, the base in read j aligned to locus k of the reference genome is a sequencing error. The true base can be the reference one or the SNV. Thus, we can use the error rate and the nucleotide frequencies observed at the locus k, to estimate the probability of read j supporting this SNV event. **In case 4**, if the alignment between read j and the reference genome does not cover locus k (missing information), we use the nucleotide frequencies observed at the locus k to estimate the probability of read j supporting this SNV event.

Similarly, we define $r_k^{(j)}$ as the "reference probability" that read j supports the reference base at locus k. And we can use the equation (2) to calculate $r_k^{(j)}$.

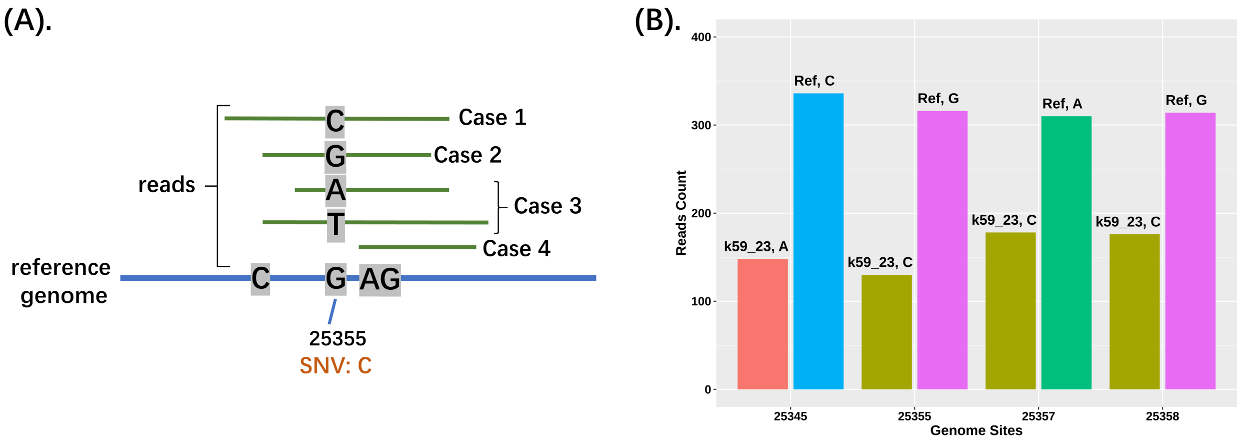

Supplementary Figure S4. A. Four cases for equation (1) at locus 25355. B. The read count comparison matching the reference and k59_23 at the four SNV sites, respectively.

$$r_k^{(j)} = \begin{cases} 10^{-\frac{q_k^{(j)}}{10}} & \text{if } q_k^{(j)} \neq 0 \text{ } and \text{ } c_k^{(j)} = 1 \\ 1 & \text{if } q_k^{(j)} = 0 \text{ } and \text{ } c_k^{(j)} = 1 \\ 10^{-\frac{q_k^{(j)}}{10}} \times (1 - \frac{m_k}{d_k}) & \text{if } q_k^{(j)} \neq 0 \text{ } and \text{ } c_k^{(j)} = 0 \\ 1 - \frac{m_k}{d_k} & \text{if } q_k^{(j)} = 0 \text{ } and \text{ } c_k^{(j)} = 0 \end{cases} \tag{2}$$

Based on the SNV probability equation (1) and reference probability equation (2), we define Equation (3) to calculate the probability that read j is consistent with haplotype i, where i is one of the 15 SNV combinations.

$$Pr(H^{(i)}|R^{(j)}) = \prod_{k=1:4, h_k^{(i)}=1} h_k^{(i)} p_k^{(j)} \times \prod_{k=1:4, h_k^{(i)}=0} (1 - h_k^{(i)}) r_k^{(j)} \tag{3}$$

$R^{(j)}$ is the read j. $H^{(i)} = \{h_k^{(i)} | k = 1, ..., 4\}$ is the haplotype i. $h_k^{(i)} \in \{0, 1\}$ is an indication function such that $h_k^{(i)} = 1$ if haplotype i contains the SNV at locus k and $h_k^{(i)} = 0$ if the haplotype i contains the reference base at locus k (e.g. the indication function for haplotype AGCG will be $\{1, 0, 1, 0\}$). To find out the most possible haplotype that is different from the reference genome, we use Equation (4) to calculate the normalized weight of each haplotype based on the read mapping quality of a set of given reads. This weight denotes the consistency between each haplotype and the set of reads.

$$Weight(H^{(i)}|R) = \frac{\sum_{R^{(j)} \in R} (1 - 10^{-\frac{Q_{map}^{(j)}}{10}}) Pr(H^{(i)}|R^{(j)})}{\sum_{R^{(j)} \in R} (1 - 10^{-\frac{Q_{map}^{(j)}}{10}})} \tag{4}$$

where $Q_{map}^{(j)}$ is the mapping quality of read j obtained from bowtie2 [7]. $(1 - 10^{-\frac{Q_m^{(j)}ap}{10}})$ denotes the estimated probability that the alignment of read j corresponds to the true region. R is a set of reads that cover and match at least one of the four loci of the reference genome we mentioned above. Table S7 shows the weights of different haplotypes.

We can see that the haplotype **ACCC** at four loci is the most possible one of the fifteen possible haplotypes. Its weight is about 0.2235, which is about the twice of the second-highest weight. Thus we add this possibly novel haplotype into the reference database and used VirStrain to search for this haplotype in other Washington State samples.

In order to validate this finding, we checked whether other samples also contain k59_23. Towards this goal, we added this strain to the VirStrain database and then used VirStrain to detect it in other SRA datasets from Washington State. The output shows that k59_23 was also detected in three other Washington State samples.

Supplementary Table S7. Weights of different haplotypes. The character with blod face is a SNV.

Haplotype	Weight	Haplotype	Weight	Haplotype	Weight
**ACCC**	0.2235	**AC**A**C**	0.0799	CGA**C**	0.0321
C**CCC**	0.1258	**A**GAG	0.0499	**AC**AG	0.0284
**A**G**CC**	0.1160	**A**GA**C**	0.0497	C**C**A**C**	0.0236
CG**CC**	0.0816	**ACC**G	0.0383	C**CC**G	0.0200
**A**G**C**G	0.0815	CG**C**G	0.0323	C**C**AG	0.0160

The VirStrain detection results of these four data sets are summarized in Supplementary Table S8. Both the statistical analysis and the VirStrain outputs show that a minor strain likely exists in these samples. This indicates the possible utility of VirStrain in detecting multi-infection cases.

BioSample accession number	VirStrain Result 1	Relative abundance 1	VirStrain Result 2	Relative abundance 2
SAMN15678406	MT641500.1	0.8523	k59_23	0.1477
SAMN15678405	MT375468.1	0.9042	k59_23	0.0958
SAMN15678404	MT632835.1	0.8797	k59_23	0.1203
SAMN15678403	MT628075.1	0.9006	k59_23	0.0994

Supplementary Table S8. The identification results of VirStrain of four Washington State samples. VirStrain can identify two strains in these data sets. The numbers "1" and "2" represent these 2 strains. The major strains in these four samples returned by VirStrain are different but all have geographical locations from Washington State. The relative abundance of each strain is calculated by normalizing the depth of all identified strains.

1.10 VirStrain predicts HIV subtype and H1N1 clade

The identification result generated by VirStrain can also be used to extract the subtype or clade information of identified strains. Thus, users can obtain the strain subtype or clade information from reads directly without assembly, which is useful for datasets that cannot form quality assemblies. In this experiment, we applied VirStrain to four real datasets and output the subtype or clade information of identified strains using a bottom-to-up method. To know the subtype or clade type of the input dataset, we can check the metadata of "the most possible strain" output by VirStrain. Then, we use the subtype or clade label of the identified strain as the final output.

Supplementary Table S9 shows that the subtype or clade identified by VirStrain is the same as the truth. Besides, the region information is also consistent with the source region of these samples.

BioSample accession number	Virus type	Subtype/Clade (Truth)	Subtype/Clade (VirStrain)	Region of sample	Major region of cluster
SAMN10225085	HIV	F1	F1	Brazil	Brazil
SAMN08101778	HIV	B	B	Brazil	Brazil
SAMN01095441	H1N1	1A.3.3.2	1A.3.3.2	Hong Kong	Hong Kong
SAMN02719578	H1N1	Other-Human	Other-Human	Unknown	Russia

Supplementary Table S9. The VirStrain clade/Subtype prediction result of 4 real sequencing data. "Subtype": the subtype of HIV strain is obtained according to literature [8]. "Clade": the clade of H1N1 strain is estimated by the Swine H1 clade classification tool of Influenza Research Database (https://www.fludb.org).

1.11 Limitation posed by iterative search

As many reference strains share high sequence similarity, after finding the best match in the reference genomes, VirStrain will remove the matched SNV sites before the next iteration so that other possible strains with different SNV sites can achieve better $Vscore$. Without removing the matched SNV sites, strains that share high similarity with the identified strain will naturally returned as other possible strains, incurring false positive identifications. However, there

is a tradeoff between the false positive identification and sensitivity. When the SNV sites of the best matched strain are removed, the strains that are highly similar to the best matched one may be missed by VirStrain. To investigate this, we constructed a difficult case where three strains with different similarities co-exist in one sample and tested the performance of VirStrain.

Thus, the iterative search procedure poses a constraint on the number of different SNVs between strains in the same sample. Strains with too few differences will be missed by VirStrain. In order to provide the guidance on the number of expected different SNVs, we tested a hard case for our method. The input data contain reads from three strains, with one being the major one (100x) and other two being minor ones (10x). In addition, two of the three strains are highly similar with only 1 different SNV while the rest one has more than 3 different SNVs (strains with 3, 5 and at least 10 different SNVs were used and considered as 3 groups in this experiment). In total, there are 30 simulated datasets and 10 simulated datasets in each group. Supplementary Table S10 shows VirStrain is always able to identify strains with high abundance and more than 3 different SNVs but misses the strain with 1 different SNV. In addition, we found the median number of best matches decrease with the increase of the number of different SNVs. Based on these results, we recommend users to consider 10 as a minimum number to identify other possible strains in multi-strain infection cases. It should be noted this value may increase for viruses with large genome sizes. For example, we take 150 as the threshold for HCMV. For low abundance strains identified in all tie cases, VirStrain will output multiple best matches in descending order of $Vscore$ of the first iteration. We found the correct strain was always the top 1 strain under this sorting rule, which could be a useful information for users to identify the correct strain among multiple best matches.

Group	Depth	Identification result	Tie cases	Median number of best matches
(H, 1, 3)	(100X, 10X, 10X)	(10, 0, 10)	(0, 0, 4)	(1, 0, 6)
(H, 1, 5)	(100X, 10X, 10X)	(10, 0, 10)	(0, 0, 4)	(1, 0, 5)
(H, 1, >=10)	(100X, 10X, 10X)	(10, 0, 10)	(0, 0, 4)	(1, 0, 2)

Supplementary Table S10. The performance of VirStrain on 30 3-strain simulted datasets with different settings (10 simulated datasets in each group). “H” in “Group” column represents the high abundance strain, and other two values in the tuple represent the strain with x SNVs (x refers to the value in the tuple). For “Identification result”, “Tie cases”, “Median number of best matches” columns, each value in the tuple represents the count of these attributes for correspond strain among all simulated datasets of that group. For example, “(10, 0, 10)” in the first row, “Identification result” column represents high abundance strain and the strain with 3 different SNVs can be identified in all 10 datasets while the strain with 1 different SNV is lost in all tested datasets.

1.12 Properties comparison between different tools

Tools	Type	Strain profiles	Interactive report	SNV frequency information	Require N	Computational efficiency
VirStrain	Identification tool	✓	✓	✓	×	COVID19-R1 (19.5G): 215s<br>HCMV-M1 (2.0G): 27s<br>HCMV-R1 (9.4G): 62s
Centrifuge	Identification tool	✓	×	×	×	COVID19-R1 (19.5G): 290s
Krakenuniq	Identification tool	✓	×	×	×	COVID19-R1 (19.5G): 254s
Sigma	Identification tool (Alignment-based)	✓	×	×	×	COVID19-R1 (19.5G): >15h
Pathoscope2	Identification tool (Alignment-based)	✓	×	×	×	COVID19-R1 (19.5G): 1721s
TenSQR	Haplotype reconstruction tool	✓	×	×	×	HCMV-R1 (9.4G): >7 days using 8 threads
HaROLD	Haplotype reconstruction tool	✓	×	✓	✓	HCMV-M1 (2.0G): 3h 24min<br>HCMV-R1 (9.4G): 690s
CliqueSNV	Haplotype reconstruction tool	✓	×	×	×	HCMV-R1 (9.4G): >7 days using 8 threads
aBayesQR	Haplotype reconstruction tool	✓	×	×	×	HCMV-R1 (9.4G): >7 days using 8 threads
PredictHaplo	Haplotype reconstruction tool	×	×	×	✓	HCMV-M1: 10 days using 24 threads [9]
ShoRAH	Haplotype reconstruction tool	×	×	✓	×	HCMV-M1: 10 days using 24 threads [9]

Supplementary Table S11. Properties of existing tools. "✓" indicates the software has the feature, otherwise, it is "×"; "Require N" indicates whether a tool requires users' inputs about the number of haplotypes. All the experiments for the "Computational efficiency" are tested on an HPCC CentOS 6.8 node with 2.4Ghz 14-core Intel Xeon E5-2680v4 CPUs and 128 GB memory. We use 8 threads for all tools. For all tested datasets in "Computational efficiency", the NCBI SRA accession IDs are listed as follows: COVID19-R1: SRR10971381; HCMV-M1: ERR3276894; HCMV-R1: SRR11053571.

2 Supplementary Methods

2.1 *k*-mer extraction from SNV sites

We will extract k-mers from these SNV sites, with the center base of each k-mer coming from this site. The complete k-mer is then constructed by including $\frac{k-1}{2}$ bases to the left and right side of a variation site. Supplementary Figure S1 shows an example of k-mer extraction.

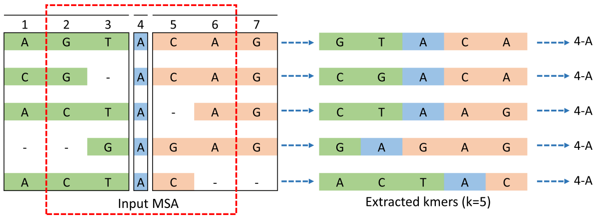

Supplementary Figure S7. The process of k-mer extraction. Given the chosen site, k-mers are extracted from the centering base and neighbouring sequences.

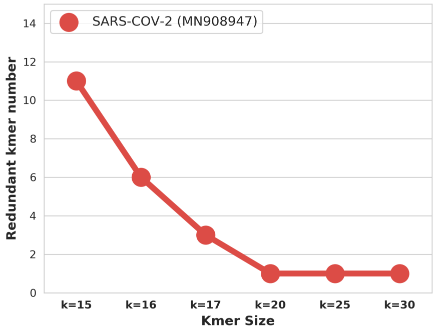

Supplementary Figure S8. The relationship between k-mer size and the number of redundant k-mers. When $k \geq 20$, the only repetitive k-mer is a repeat of “A”, which is not used in our method.

If k-mers repeat at different sites in the reference genomes, false positive strain identification will happen. Intuitively, bigger k-mers tend to appear less frequently than small k-mers. We thus investigate how many times we will observe the same k-mer at different sites in an MSA. As the reference genomes SARS-CoV-2 have high sequence similarity and larger genome sizes than other known RNA viruses, we conducted this experiment on SARS-CoV-2. The results are summarized in Supplementary Figure S2. As the figure shows, with the increase of k, the repeat numbers of the k-mer at different sites reduce quickly. Thus, either large k can be chosen or we can directly exclude those columns that contain repetitive k-mers in order to fulfill the assumption.

3 Supplementary Figures

A.

Strain ID	Cls info	SubCls info	Vscore	Valid map rate	Site coverage	Strain depth	Strain info	SNV freq
>> Most possible strains								
>MT451123.1	Cluster2830_2	NA	1	2877/2877	2877/2877	73.62872975	Name:AUS/VIC223/2020|Clade:19A	{'574-G': 97, '592-G': 57,...}
>> Other possible strains								
Can not detect other strains.								
>> Highest_Map_Strains (Could be FP):								
>MT451123.1	Cluster2830_2	NA	1	2877/2877	2877/2877	73.62872975	Name:AUS/VIC223/2020|Clade:19A	{'574-G': 97, '592-G': 57,...}
>> Top10_Score_Strains:								
>MT451123.1	Cluster2830_2	NA	1	2877/2877	2877/2877	NA	Name:AUS/VIC223/2020|Clade:19A	NA
>MT641682.1	Cluster950_2	Not_record	0.9996053	2876/2877	2876/2877	NA	Name:AUS/VIC509/2020|Clade:19A	NA
>MT451218.1	Cluster2887_3	Not_record	0.999361	2875/2877	2875/2877	NA	Name:AUS/VIC326/2020|Clade:19A	NA
>MT451197.1	Cluster2879_1	Not_record	0.9990321	2874/2876	2874/2876	NA	Name:AUS/VIC305/2020|Clade:19A	NA
>MT633044.1	Cluster1615_3	Not_record	0.9989005	2874/2877	2874/2877	NA	Name:USA/WA-S413/2020|Clade:19A	NA
>MT451079.1	Cluster2812_1	Not_record	0.9988911	2874/2876	2874/2876	NA	Name:AUS/VIC176/2020|Clade:19A	NA
>MT407654.1	Cluster2152_2	Not_record	0.9986045	2873/2877	2873/2877	NA	Name:CHN/OS1/2020|Clade:19A	NA
>MT459850.1	Cluster2619_2	Not_record	0.9985481	2873/2877	2873/2877	NA	Name:GRC/134_32124/2020 |Clade:19A	NA
>MT358709.1	Cluster3485_2	Not_record	0.9985481	2873/2877	2873/2877	NA	Name:USA/ID-UW-3892/2020|Clade:19A	NA
>MT451220.1	Cluster2888_2	Not_record	0.9985434	2873/2877	2873/2877	NA	Name:AUS/VIC328/2020|Clade:19A	NA

B.

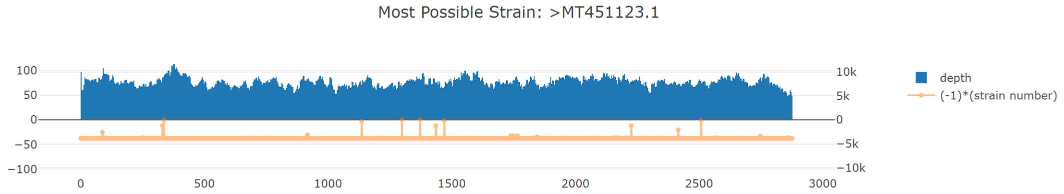

Supplementary Figure S5. A. The VirStrain text report of one SARS-CoV-2 simulated dataset (Truth: MT451123.1). The detailed explanation of the columns can be found at the Github website of VirStrain. B. The VirStrain html report of one SARS-CoV-2 simulated dataset (Truth: MT451123.1). X-axis refers to all selected sites by the greedy covering algorithm. The top Y-axis represents the predicted sequencing depth of each site. The bottom Y-axis represents the number of strains sharing this site, which reflects the site uniqueness.

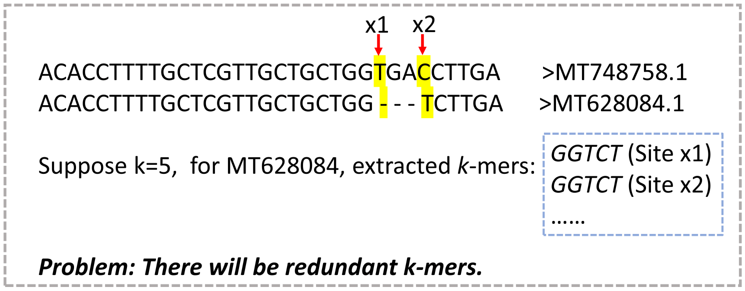

Supplementary Figure S6. An example showing the problem caused by dash.

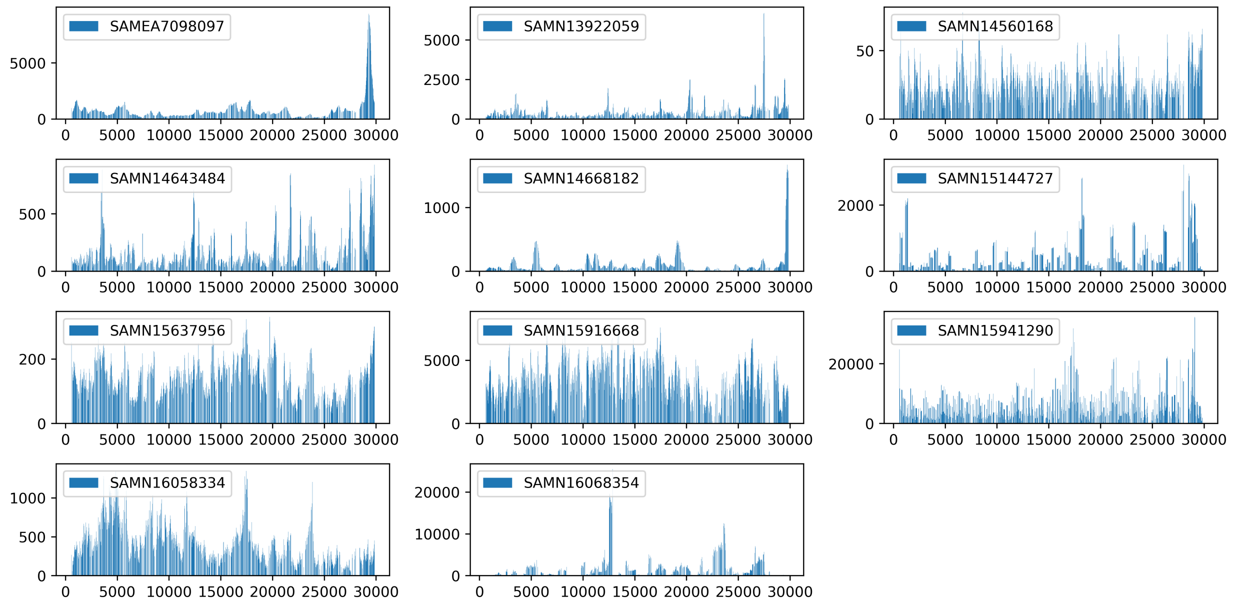

Supplementary Figure S9. The sequencing depth distribution of the SNV sites of SARS-CoV-2 genomes in 11 real sequencing datasets. X-axis represents the position of identified genomes. Y-axis represents the sequencing depth of chosen SNV sites by the greedy covering algorithm. The strains shown in these figures are all correctly identified.

References

[1] Y. Shu and J. McCauley. GISAID: Global initiative on sharing all influenza data - from vision to reality. *Euro Surveill*, 22(13), 2017.

[2] J. Hadfield et al. Nextstrain: real-time tracking of pathogen evolution. *Bioinformatics*, 34(23):4121–4123, 2018.

[3] Z. Zhang et al. A greedy algorithm for aligning DNA sequences. *J. Comput. Biol.*, 7(1-2):203–214, 2000.

[4] M. Abdou Chekaraou et al. A novel hepatitis B virus (HBV) subgenotype D (D8) strain, resulting from recombination between genotypes D and E, is circulating in Niger along with HBV/E strains. *J Gen Virol*, 91(Pt 6):1609–1620, 2010.

[5] Y. Hu et al. Identification of Two New HIV-1 Circulating Recombinant Forms (CRF87_cpx and CRF88_BC) from Reported Unique Recombinant Forms in Asia. *AIDS Res Hum Retroviruses*, 33(4):353–358, 2017.

[6] D. Li et al. MEGAHIT: an ultra-fast single-node solution for large and complex metagenomics assembly via succinct de Bruijn graph. *Bioinformatics*, 31(10):1674–1676, 2015.

[7] Ben Langmead and Steven L Salzberg. Fast gapped-read alignment with bowtie 2. *Nature methods*, 9(4):357, 2012.

[8] B. M. Alves et al. Estimating HIV-1 Genetic Diversity in Brazil Through Next-Generation Sequencing. *Front Microbiol*, 10:749, 2019.

[9] Z. L. Deng, A. Dhingra, et al. Evaluating assembly and variant calling software for strain-resolved analysis of large DNA viruses. *Briefings in Bioinformatics*, 22(3), 2021.
