# Supplementary material for: VirStrain: a strain identification tool for RNA viruses
Source: Genome Biol. 2022 Jan 31;23:38. doi: 10.1186/s13059-022-02609-x (PMC8801933; doi:10.1186/s13059-022-02609-x)
Supplement: Supplementary file 2 — Additional file 2 Review history. [file 13059_2022_2609_MOESM2_ESM.docx]

**1^st^ round**

**Reviewer 1**

The manuscript by Liao et. al. titled "VirStrain: a strain identification tool for RNA viruses" present a new reference-based software for the identification of viral strains. At least since the start of the global SARS-CoV-2 pandemic, it is clear that identifying viral strains is a relevant and important task.

The paper is well written and describes the algorithm and evaluation in a clear way. The software itself is easily installed, useable out of the box and fast. Tests on own data sets seem to confirm the performance described in the article. Especially the identification and abundance estimates in samples with multiple closely related strains is impressive.

The data sets used for evaluation are sound and some of them are already used in the community. Of course VirStrain, like all reference-based methods, is dependent on accurate and extensive data bases which might be the case for viruses like SARS-CoV-2 or HIV. In particular regarding bacterial data sets as described in the conclusion, the completeness and bias in databases will play a major role though and should be mentioned.

Finally, since the algorithm is k-mer/SNV-based, it is not clear how it would react in the presence of recombination events, prevalent in viruses like Influenza or HCMV. The authors describe a data set with Influenza but seem to focus on the HA region of H1N1. There should be some evaluation whether VirStrain is able to tell recombinant viruses apart or a description of the limitations with regard to recombination events.

Further comments:

Figure 4 should either be removed or the differences made more visible. In the current form there is not much to learn from the figure.

**Reviewer 2**

The paper describes a new tool called VirStrain, which can identify strains of a virus within an NGS sample. Overall, unfortunately, I don't think this paper is significant enough for Genome Biology.

I had a few issues with the paper in general.

1) The SARS-CoV-2 numbers and data used are well, well out of date, well over a year out of date - this needs to be updated

2) The introduction was, in my opinion, not really setting the correct scene for VirStrain, it does not introduce the vast amount of VIRAL haplotype reconstruction tools (the classics being ShoRAH, QuRe and PrecitHaplo but there are vast amounts of newer ones) which can identify strains. There are other tools as well such as SHIVER for HIV (which is one of the viruses used here) which analyse the reads to select the most appropriate reference sequence. More importantly, it does not focus on NGS reads, it seems to focus on sequences on GISAID etc, and molecular epidemiology techniques which rely on the full genome. I don't think virStrain helps there, it is more of a mixed strain identification tool.

3) I don't think SARS-CoV-2 is really a very good example to use at all. It has slowly evolved away from the original Wuhan sequence. You can simply use that as the reference, align the reads, and call a consensus - to get the sequence of your sample - there is no real need to find "a best" reference sequence. This does no address mixed infection, but haplotype reconstruction could be used as could plain old variant frequencies.

4) For the hamming distance plots of the strains - Figure 1 - you should check out tools such as PASC and Demarc which do similar things like this - these should be referenced/checked out

Overall, I just found it a little confused. It seems that the primary aim is the identification of mixed strain infection, but a lot of the focus is on molecular epidemiology and throughout in the introduction, and I don't think the tools being compared to are the right ones. In addition, if the sample is "new" - then it will likely be slightly different from anything seen before - so you want to know the exact sequences of the two strains, not simply what it is closest to.

I would suggest completely focussing on the mixed strain identification of VirStrian, it's use on NGS reads. I think the molecular epidemiology slant (and also the SARS-CoV-2) angle do not help really.

There is also a tools called HAROLD - which is a mixed strain identifier based on NGS reads (as well as a few more), I think VirStain needs to be compared to these, and viral haplotype reconstruction algorithms in general. If the focus is not on getting the "full genome" of each strain out, then best to focus on what just knowing something is mixed tells you.

You have the makings of a nice tool, but I think you need to focus down on case studies that really show its worth –

**Reviewer 3**

Liao et al. present VirStrain, a new kmer based tool that can predict which reference haplotypes are present in a library of short reads. The authors focus their validations and applications using databases of RNA viruses.

Major comments

I found it strange that the tool is called VirStrain and that the title is even more specific to RNA viruses. Are there any limitations that prevent application of the method to DNA viruses or bacteria? Is there anything about the method that makes it specific or optimized for RNA viruses? How does the method handle longer genomes (like from bacteria or giant viruses)?

Table 1 clearly indicates VirStrain has poor performance for the SARS-CoV-2 simulation when sequencing depth is below 20x. The authors should evaluate other methods (Sigma, Pathoscope, Centrifuge, Kraken) on these same datasets (or simulated ones with variable coverage). Do other methods outperform VirStrain on low coverage samples? Also please add details about the dataset in the table 1 legend.

I may have missed it, but is VirStrain accurate when an RNA virus is present in a complex community with other viruses and bacteria?

The authors should follow best-practices for distributing their software, including using anaconda and versioned releases. Compatibility with docker would also be a plus.

I wonder how the number of reference genomes affects performance. I might expect that more sequencing coverage is needed when the size of the database gets very large in order to distinguish between many different possible reference haplotypes. Is this the case? For example, going back to the table 1 data, would VirStrain have better performance at lower coverage if there were fewer SARS-CoV-2 genomes in the database?

Minor comments

In the discussion the authors say that VirStrain has poor performance for "<10x". this should be changed to "<20x".

For figure 4 is it difficult to see the differences between methods. For visualization please truncate the y-axis at 0.75, or add hash marks // to indicate a gap in the y-axis from 0.0 to 0.75.

Please include github URL in abstract

Ran into errors during software testing:

$ python VirStrain.py -i Test_Data/MT451123_1.fq -d VirStrain_DB/SCOV2 -o MT451123_SE_Test

Txt report is done. Now will generate pdf report!

Traceback (most recent call last):

File "bin/S4_Plot_strain_cov.py", line 6, in <module>

from _plotly_future_ import v4_subplots

ModuleNotFoundError: No module named '_plotly_future_'

mv: cannot stat 'VirStrain_report.html': No such file or directory

**Authors’ response**

Reviewer 1:

- The manuscript by Liao et. al. titled "VirStrain: a strain identification tool for RNA viruses" present a new reference-based software for the identification of viral strains. At least since the start of the global SARS-CoV-2 pandemic, it is clear that identifying viral strains is a relevant and important task.

The paper is well written and describes the algorithm and evaluation in a clear way. The software itself is easily installed, useable out of the box and fast. Tests on own data sets seem to confirm the performance described in the article. Especially the identification and abundance estimates in samples with multiple closely related strains is impressive.

The data sets used for evaluation are sound and some of them are already used in the community.

*We thank the reviewer for the positive feedback and are delighted to know that our work is helpful.*

- Of course, VirStrain, like all reference-based methods, is dependent on accurate and extensive data bases which might be the case for viruses like SARS-CoV-2 or HIV. In particular regarding bacterial data sets as described in the conclusion, the completeness and bias in databases will play a major role though and should be mentioned.

*We thank the reviewer for this* *thoughtful suggestion and agree that the completeness and bias of the reference genome database can affect reference-based methods including VirStrain. Because VirStrain allows the users to provide their own reference genome database, users can add more reference genomes to keep up with the extensive sequencing efforts for viruses. Meanwhile, we also provided two pre-built reference databases at the github repository. The first one is the database we used in this paper. The second one is newly constructed before the submission of the revision, containing 6,880 strains covering over 20 SARS-CoV-2 clades from December 2019 to October 2021.*

*The bias is hard to fix by VirStrain. We explained in the Discussion section that the users can conduct pre-processing to mitigate the data contamination or bias problem. Please find the revision in the Discussion Section (Page 21).*

- Finally, since the algorithm is k-mer/SNV-based, it is not clear how it would react in the presence of recombination events, prevalent in viruses like Influenza or HCMV. The authors describe a data set with Influenza but seem to focus on the HA region of H1N1. There should be some evaluation whether VirStrain is able to tell recombinant viruses apart or a description of the limitations with regard to recombination events.

*We thank the reviewer for this comment. For segmented viruses, our method currently conducts multiple sequence alignment for each segment separately and thus it cannot detect reassortment between the segments. For recombination within the same segment, we did not exclude any reference strains with recombination. If the recombinant viruses still possess SNVs, they can still be identified. We tested whether VirStrain can identify recombinant strains from mix-strain infections in this revision. The result of our experiment shows that VirStrain is still able to identify the recombinant HIV and HBV strains from simulated mix-strain sequencing data (Supplementary Section 2.5 on page 5, Table S4). However, suppose that strain C is a recombination of the parent strains A and B, and C does not possess any other mutations/variants, the k-mers corresponding to the recombination event are all clustered at one loci in C. In this case, VirStrain will not be able to separate C from A and B. We added the discussion in Section 2.5 of the main article (page 11).*

- Figure 4 should either be removed or the differences made more visible. In the current form, there is not much to learn from the figure.

*We thank the reviewer for pointing this out. We have truncated the y-axis at 0.7 to make it more visible.*

Reviewer 2:

- The paper describes a new tool called VirStrain, which can identify strains of a virus within an NGS sample. Overall, unfortunately, I don't think this paper is significant enough for Genome Biology.

- I had a few issues with the paper in general.

1) The SARS-CoV-2 numbers and data used are well, well out of date, well over a year out of date - this needs to be updated

*We thank the reviewer for this comment. When we gave general descriptions about SARS-CoV-2 in this revision, we used the latest numbers. For the experiments, we kept using the reference database we built when we submitted the paper, which contained all the reference strains in NCBI before July 14, 2020. This helps us avoid rerunning all the experiments for all the tools, some of which can consume a large amount of computing resources. This reference database contains 4082 strains and is large enough to compare different tools. To show how the database size affects the performance of VirStrain, we added a targeted experiment that tested VirStrain on three reference databases of 3347, 4082, and 6880 strains. The 6880 strains cover over 20 SARS-CoV-2 clades from December 2019 to October 2021. More detailed information about this larger database can be found in Supplementary Section 2.1. The results show that a larger reference database can slightly increase the cases of multi-hits, meaning that multiple strains with the same ranking are returned by VirStrain. But this poses the same challenge for all the tested reference-based tools. The new experiment can be found in Supplementary Section 2.1 and 2.3. In our github website, we provided users with* *two pre-built databases containing 4082 and 6880 SARS-CoV-2 strains, respectively. Users can also provide a fasta file containing their own referfence genomes so that they can keep adding newly sequenced strain genomes.*

2) The introduction was, in my opinion, not really setting the correct scene for VirStrain, it does not introduce the vast amount of VIRAL haplotype reconstruction tools (the classics being ShoRAH, QuRe and PrecitHaplo but there are vast amounts of newer ones) which can identify strains. There are other tools as well such as SHIVER for HIV (which is one of the viruses used here) which analyse the reads to select the most appropriate reference sequence. More importantly, it does not focus on NGS reads, it seems to focus on sequences on GISAID etc, and molecular epidemiology techniques which rely on the full genome. I don't think virStrain helps there, it is more of a mixed strain identification tool.

*Thanks. We have heavily modified the Introduction and focused on strain identification from short reads. We are fully aware of the research of viral haplotype reconstruction and we contributed one tool* *[2]. We agree that viral haplotype reconstruction can be applied to strain identification. We benchmarked VirStrain against popular or recently published reference-based haplotype reconstruction tools (Section 2.5 on page 9).* *We did not include QuRe and SHIVER for the following reasons. As shown in* *a recent review about haplotype reconstruction tools [3], QuRe has the memory usage issue, which we also experienced. Thus, we cannot finish the experiments using QuRe. Also, we did not benchmark SHIVER because it produces only one custom reference sequence and thus is not applicable to multi-strain infections. It is worth noting that haplotype reconstruction tools don’t restrict the finally assembled/reconstructed sequences to the known strains like a typical strain identification tool. Thus, for a fair comparison, we should not require that the reconstructed haplotypes are identical to the strains in the sample. Instead, we check whether the reconstructed haplotypes are “close” enough to the ground truth. If there are still a large number of strains between the identified haplotypes and the ground truth strain, this indicates low quality/accuracy of haplotype reconstruction. In summary, haplotype reconstruction using short reads is a highly challenging problem. The available tools have limitations in the length of the reconstructed haplotypes, assembly accuracy, estimation of the number of the haplotypes without confidence scores, etc. They usually perform well on the commonly used 5-HIV strain benchmarking dataset because the similarity of the 5 strains is not very high, which have pairwise sequence similarities from 91.8% to 97.4% [2]. However, if the mixed strains are highly similar (such as those in our experiments), the performance of haplotype reconstruction tools often deteriorates quickly.*

*Thus, although haplotype reconstruction tools can be applied to multi-strain identification, their outputs tend to be error-prone, particularly on datasets containing highly similar strains with low abundance. VirStrain provides an alternative solution with better accuracy for known strain identification.*

*The newly added experiments on using haplotype reconstruction tools can be found in Section 2.5 on page 9.*

3) I don't think SARS-CoV-2 is really a very good example to use at all. It has slowly evolved away from the original Wuhan sequence. You can simply use that as the reference, align the reads, and call a consensus - to get the sequence of your sample - there is no real need to find "a best" reference sequence. This does not address mixed infection, but haplotype reconstruction could be used as could plain old variant frequencies.

*We thank the reviewer for this comment. We agree that read mapping and consensus derivation are sufficient to get the strain when there is only one. When there are multiple strains, haplotype reconstruction can be applied but the high similarity between haplotypes can jeopardize the accuracy of haplotype reconstruction. Newly added experiments using haplotype reconstruction tools have shown this (Section 2.5 on page 10, Figure 3 and Section 2.8.2 on page 15, Figure 5).*

*Overall, SARS-CoV-2 poses a hard case for both known strain identification and haplotype reconstruction because of the low divergence. The sheer amount of strains and their high sequence similarity is a good test case for strain identification and haplotype reconstruction tools. Many of the haplotype reconstruction tools were tested on HIV, which have much lower similarities than SARS-CoV-2. We acknowledged the traditional methods and added the reason for choosing SARS-CoV-2 in the Introduction (page 2) and Results (page 3) in this revision.*

4) For the hamming distance plots of the strains - Figure 1 - you should check out tools such as PASC and Demarc which do similar things like this - these should be referenced/checked out

*We thank the reviewer for this suggestion. These two tools are referenced in this revision (Section 2.1 on page 4).*

- Overall, I just found it a little confused. It seems that the primary aim is the identification of mixed strain infection, but a lot of the focus is on molecular epidemiology and throughout in the introduction, and I don't think the tools being compared to are the right ones. In addition, if the sample is "new" - then it will likely be slightly different from anything seen before - so you want to know the exact sequences of the two strains, not simply what it is closest to. I would suggest completely focussing on the mixed strain identification of VirStrian, it's use on NGS reads. I think the molecular epidemiology slant (and also the SARS-CoV-2) angle do not help really.

*We thank the reviewer for the suggestion. We heavily modified the Introduction section to focus on strain identification from short reads. In addition, we added three experiments to show the application of VirStrain in mixed strain infections as suggested by the reviewer. Firstly, we applied VirStrain and other tools to two real samples with the co-infection of SARS-CoV-2 strains (Section 2.8.2 on page 15). The result shows that VirStrain is the only tool that identifies all strains in two samples with accurate abundance estimation (see Figure 5). Then, we used twelve real mix-strain sequencing data with known compositions to evaluate the performance of VirStrain and other tools (Section 2.10 on page 17). In all these datasets, VirStrain can correctly identify both the dominant strain and the minor strain. For haplotype reconstruction tools, we find that the strains reconstructed by most tools can be aligned to other strains in the database with higher similarity than to the ground truth (see Supplementary Table S3). Besides, for HaROLD, we find that it tends to underestimate the number of strains (see Figure 3B, Figure 5, and Table 6). Finally, we applied VirStrain to a study cohort with mixed HCMV infections and reported some interesting observations (Section 2.11 on page 18). For example, we find that the genotypes found in the breast milk samples from five HIV-infected Kenyan mothers all contain strains (MK422176.1, MK290742.1, etc) isolated from the breast milk of HIV-infected mothers in Zambia [4]. We think these results can reflect the great potential of VirStrain for the analysis of mixed strain infections.*

- There is also a tool called HAROLD - which is a mixed strain identifier based on NGS reads (as well as a few more), I think VirStain needs to be compared to these, and viral haplotype reconstruction algorithms in general. If the focus is not on getting the "full genome" of each strain out, then best to focus on what just knowing something is mixed tells you.

*We thank the reviewer for this comment**. In this revision, we have added 6 haplotype reconstruction tools (ShoRAH, PredictHaplo, HaROLD, aBayesQR, CliqueSNV, and TenSQR) to the comparison in the multi-strain simulated data experiment (see right panel of Figure 3A). The result shows that most of these tools have a good performance on HIV while failing to reconstruct many strains of SARS-CoV-2 and H1N1, which have higher similarities between strains. Furthermore, we examined the difference between the falsely reconstructed haplotypes and the ground truth (Supplementary Figure S4). We find that* *all tested tools tend to miss the minor strain (see Figure 3B and Supplementary Table S3). In terms of abundance estimation, VirStrain also has the best performance (Figure 3B). In addition, we compare the properties of different tools in Supplementary Table S11 and find that VirStrain is the most efficient tool.*

- You have the makings of a nice tool, but I think you need to focus down on case studies that really show its worth –

*We thank the reviewer for this suggestion. More case studies about multi-strain infection have been added in this revision (Section 2.8.2, Section 2.10, Section 2.11, Supplementary Section 2.5).*

Reviewer 3:

- Liao et al. present VirStrain, a new kmer based tool that can predict which reference haplotypes are present in a library of short reads. The authors focus their validations and applications using databases of RNA viruses.

- Major comments

- I found it strange that the tool is called VirStrain and that the title is even more specific to RNA viruses. Are there any limitations that prevent application of the method to DNA viruses or bacteria? Is there anything about the method that makes it specific or optimized for RNA viruses? How does the method handle longer genomes (like from bacteria or giant viruses)?

*We thank the reviewer for this comment. We focused on RNA viruses because of two reasons. First, a large amount of strains and their high similarity calls for a dedicated strain identification tool. Second, the design of the method relies on multiple sequence alignment (MSA) between the strain genomes. The small RNA virus genomes and their high similarity allow fast and high quality MSA. As long as the strain genomes can be aligned using available MSA programs, VirStrain can also be applied to other types of viruses. In the revision, we applied VirStrain to 2 DNA viruses, HBV and HCMV with the latter being much larger than typical RNA viruses. The result shows that VirStrain still has good performance on these 2 viruses (see Section 2.10 on page 17 and 2.11 on page 18). But VirStrain is not optimized for bacteria because it is too slow to align all the bacterial strains (e.g. 1,957 Escherichia coli complete genomes in NCBI). And the larger divergence between bacterial strains also makes the quality of the alignment worse. It is our future work to extend VirStrain to bacteria.*

- Table 1 clearly indicates VirStrain has poor performance for the SARS-CoV-2 simulation when sequencing depth is below 20x. The authors should evaluate other methods (Sigma, Pathoscope, Centrifuge, Kraken) on these same datasets (or simulated ones with variable coverage). Do other methods outperform VirStrain on low coverage samples? Also please add details about the dataset in the table 1 legend.

*We thank the reviewer for this comment. We evaluated other methods (Sigma, Pathoscope2, Centrifuge, and Krakenuniq) on these same datasets (see Supplementary Section 2.3) and found that all tested tools have bad performance except Sigma. But Sigma is extremely computationally expensive. In order to run Sigma, we first needed to use a smaller reference database with 200 strains. Even with the small reference database, it took Sigma more than two weeks to analyze all datasets given 8 threads on an HPCC CentOS 6.8 node with 2.4Ghz 14-core Intel Xeon E5-2680v4 CPUs and 128 GB memory. On top of that, it has low accuracy in mix-strain identification (see Figure 3A). Also, we have added details about the dataset in the Table 1 legend.*

- I may have missed it, but is VirStrain accurate when an RNA virus is present in a complex community with other viruses and bacteria?

*We thank the reviewer for this comment. We tested whether the k-mers derived by VirStrain can match commonly seen non-viral reads or reads from other viruses in Section 2.3 (page 7). The result shows that most k-mers in the VirStrain database do not match the genomes of other species, indicating that VirStrain is not likely to mistake other species as viral strains (See Table 2). Our experiments of applying VirStrain to real viral metagenomic data in Section 2.8 (page 13) and Section 2.11 (page 18) further confirmed this.*

- The authors should follow best-practices for distributing their software, including using anaconda and versioned releases. Compatibility with docker would also be a plus.

*We thank the reviewer for this suggestion. We have added VirStrain to bioconda. Now you can install VirStrain with the command “conda install -c bioconda virstrain”. Besides, we added VirStrain to pypi, so you can install VirStrain by using “pip install virstrain==1.10” command in python. Furthermore, according to the page of bioconda, you can also try to install VirStrain using the docker container with the command “docker pull quay.io/biocontainers/virstrain:<tag>”.*

- I wonder how the number of reference genomes affects performance. I might expect that more sequencing coverage is needed when the size of the database gets very large in order to distinguish between many different possible reference haplotypes. Is this the case? For example, going back to the table 1 data, would VirStrain have better performance at lower coverage if there were fewer SARS-CoV-2 genomes in the database?

*Thanks. The expectation is consistent with our new experimental results. We built 2 reference databases with different sizes and repeated the experiment done in table 1 (see Supplementary Section 2.1). Compared to Table 1, VirStrain does have a small improvement at lower coverage when there are fewer COVID19 strains in the database.*

- Minor comments

- In the discussion the authors say that VirStrain has poor performance for "<10x". this should be changed to "<20x".

*We thank the reviewer for catching this, and we have changed this part in the main article (Page 7 line 9).*

- For figure 4 is it difficult to see the differences between methods. For visualization please truncate the y-axis at 0.75, or add hash marks // to indicate a gap in the y-axis from 0.0 to 0.75.

*We thank the reviewer for pointing this out. We have truncated the y-axis at 0.7 to make it more visible.*

- Please include github URL in abstract

*We thank the reviewer for this comment. We have included the Github URL and Zenodo link of VirStrain in “Availability of data and materials” according to the requirement of the journal.*

- Ran into errors during software testing:
$ python VirStrain.py -i Test_Data/MT451123_1.fq -d VirStrain_DB/SCOV2 -o MT451123_SE_Test
Txt report is done. Now will generate pdf report!
Traceback (most recent call last):
File "bin/S4_Plot_strain_cov.py", line 6, in
from _plotly_future_ import v4_subplots
ModuleNotFoundError: No module named '_plotly_future_'
mv: cannot stat 'VirStrain_report.html': No such file or directory

*We thank the reviewer for this comment. This error is caused by the wrong version of the python package “Plotly”. You can fix this problem by installing the recommended version V3.10.0 according to the GitHub page of VirStrain. Besides, you can also install VirStrain using bioconda, which will install all dependencies for you automatically.*

*References*

[1]. *Samoilov, A.E., Kaptelova, V.V., Bukharina, A.Y. et al. Case report: change of dominant strain during dual SARS-CoV-2 infection. BMC Infect Dis 21, 959 (2021).*
[2]. *Chen J, Zhao Y, Sun Y. De novo haplotype reconstruction in viral quasispecies using paired-end read guided path finding. Bioinformatics. 2018 Sep 1;34(17):2927-2935.*
[3]. *Eliseev, A., Gibson, K.M., et al.* *Evaluation of haplotype callers for next-generation sequencing of viruses. Infect Genet Evol 82, 104277 (2020).*
[4]. *Suárez, N.M., et al.* *Multiple-Strain Infections of Human Cytomegalovirus With High Genomic Diversity Are Common in Breast Milk From Human Immunodeciency Virus-Infected Women in Zambia. J Infect Dis 220(5),792-801 (2019).*

**2^nd^ round**

**Reviewer 1**

The points raised in the first revision were answered in a reasonable manner. The additional evaluation for DNA viruses is insightful. On page 11 there is a typo (TenSQ instead of TenSQR).

**Reviewer 2**

Overall, the reviewers have addressed all of my comments. I only have a couple of minor suggestions: If really didn't follow what Figure 3B was showing, I think the legend could do with improving to better describe what the circle sizes etc are and what this figure actually shows Figures - Not a fan of using Acc to represent Accuracy - prob better to just use Accuracy

**Reviewer 3**

I have no further comments
